# Supplementary material for: Developing a web-based patient decision aid for gastrostomy in motor neuron disease: a study protocol
Source: BMJ Open. 2019 Dec 18;9(12):e032364. doi: 10.1136/bmjopen-2019-032364 (PMC6937039; doi:10.1136/bmjopen-2019-032364)
Supplement: Author's manuscript [file bmjopen-2019-032364.draft_revisions.pdf]

BMJ Open is committed to open peer review. As part of this commitment we make the peer review history of every article we publish publicly available.

When an article is published we post the peer reviewers' comments and the authors' responses online. We also post the versions of the paper that were used during peer review. These are the versions that the peer review comments apply to.

The versions of the paper that follow are the versions that were submitted during the peer review process. They are not the versions of record or the final published versions. They should not be cited or distributed as the published version of this manuscript.

BMJ Open is an open access journal and the full, final, typeset and author-corrected version of record of the manuscript is available on our site with no access controls, subscription charges or pay-per-view fees (http://bmjopen.bmj.com).

If you have any questions on BMJ Open's open peer review process please email info.bmjopen@bmj.com

BMJ Open

DEVELOPING A WEB-BASED PATIENT DECISION AID FOR GASTROSTOMY IN MOTOR NEURONE DISEASE: A STUDY PROTOCOL

Journal:	*BMJ Open*
Manuscript ID	bmjopen-2019-032364
Article Type:	Protocol
Date Submitted by the Author:	14-Jun-2019

Complete List of Authors:	Maunsell, Rose; University of Southampton, Health Sciences<br>Bloomfield, Suzanne; Countess Mountbatten House<br>Erridge, Clare; Southampton General Hospital<br>Foster, Claire; University of Southampton, Health Sciences<br>Hardcastle, Maggi; Rowans Hospice<br>Hogden, Anne; University of Tasmania, Australian Institute of Health Service Management<br>Kidd, Alison; University of Southampton, Carer representative<br>Lisiecka, Dominika; University of Limerick, School of Allied Health<br>McDermott, Christopher; university of sheffield,<br>Morrison, Karen; University of Southampton, Faculty of Medicine<br>Recio-Saucedo, Alejandra; University of Southampton<br>Rickenbach, Louise; Motor Neurone Disease Association<br>White, Sean; Sheffield Teaching Hospitals NHS Foundation Trust, Dietetic Department<br>Williams, Peter; University of Southampton, Patient representative<br>Wheelwright, Sally J.; Univ Southampton, Health Sciences

Keywords:	Motor neurone disease < NEUROLOGY, NUTRITION & DIETETICS, Adult palliative care < PALLIATIVE CARE, Protocols & guidelines < HEALTH SERVICES ADMINISTRATION & MANAGEMENT

SCHOLARONE™
Manuscripts

DEVELOPING A WEB-BASED PATIENT DECISION AID FOR GASTROSTOMY IN MOTOR NEURONE DISEASE: A STUDY PROTOCOL

Corresponding Author

Dr Sally Wheelwright
Health Sciences,
University Of Southampton,
SO17 1BJ
Email: S.J.Wheelwright@soton.ac.uk
Tel: 02380 597860

Co-authors

Miss Rose Evill
Health Sciences,
University Of Southampton,
SO17 1BJ
Email: r.evill@soton.ac.uk
Tel: 02380 597939

Ms Suzanne Bloomfield
Community CNS in Palliative Care Team
Countess Mountbatten Hospice
Southampton, SO30 3JB
Tel: 02380 475874
Email: suzanne.bloomfield@uhs.nhs.uk

Ms Clare Erridge
Lead Motor Neurone Disease Care Centre Coordinator Mailpoint 101 Wessex Neurological Centre
Southampton General Hospital
Tremona Road
Southampton, SO16 6YD
Tel: 02381 206544
Email: clare.erridge@uhs.nhs.uk

Ms Maggi Hardcastle
Clinical Specialist Physiotherapist
The Rowans Hospice
Purbrook Heath Road
Waterlooville, PO7 5RU
Tel: 02392 250001
Email: maggi.hardcastle@rowanshospice.co.uk

Dr Anne Hogden
Senior Lecturer
Australian Institute of Health Service Management
University of Tasmania, Rozelle Campus
Cnr Glover and Church Streets, Lilyfield, NSW 2040
Tel: +61 2 8572 7922
Email: anne.hogden@utas.edu.au

Dr Alison Kidd
Lay person

For peer review only

Corlan, Pencelli, Brecon, Powys, LD3 7LX
Tel: 091874 665401
Email: alison@prospectory.co.uk

Dr Dominika Lisiecka
Lecturer in Speech and Language Therapy
School of Allied Health
Health Sciences Building
Room H2-021
University of Limerick
Email: dominika.lisiecka@ul.ie

Professor Christopher McDermott
Professor of Translational Neurology / Consultant Neurologist
Sheffield Institute for Translational Neuroscience
385a Glossop Rd
Sheffield, S10 2HQ
Tel: 0114 2222295 (Secretary)
Email: c.j.mcdermott@sheffield.ac.uk

Professor Karen Morrison
Associate Dean for Education and Professor of Neurology
Faculty of Medicine, University of Southampton
Room AB217, Level B, South Academic Block, MP801, Southampton General Hospital, SO16 6YD
Tel: 02381 208134
Email: K.E.Morrison@soton.ac.uk

Dr Alejandra Recio-Saucedo
Research Fellow, Portfolio Insight and Publications
NIHR Evaluation, Trials and Studies Coordinating Centre (NETSCC)
University of Southampton
Alpha House
Enterprise Road
Southampton, SO16 7NS
Tel: 02380 595606
Email: ars@ecs.soton.ac.uk

Ms Louise Rickenbach
Regional Care Development Adviser for South Central
Motor Neurone Disease Association
Tel: 03453751831
Email: louise.rickenbach@mndassociation.org

Mr Peter Williams
Lay Person
Corlan, Pencelli, Brecon, Powys, LD3 7LX
Tel: 091874 665401
Email: peter@prospectory.co.uk

Mr Sean White
Dietetic Department
Nurses Home
Northern General Hospital
Herries Road
Sheffield S5 7AU

For peer review only

Tel: 0114 2714162
E-mail: sean.white@sth.nhs.uk

Word count, excluding title page, abstract, references, figures and tables: 3474

DEVELOPING A WEB-BASED PATIENT DECISION AID FOR GASTROSTOMY IN MOTOR NEURONE DISEASE: A STUDY PROTOCOL

Rose Evill[1], Suzanne Bloomfield[2], Clare Erridge[3], Claire Foster[1], Maggi Hardcastle[4], Anne Hogden[5], Alison Kidd[6], Dominika Lisiecka[7], Christopher J McDermott[8], Karen Morrison[9], Alejandra Recio-Saucedo[10], Louise Rickenbach[11], Sean White[12], Peter Williams[13] and Sally Wheelwright[1]

[1]Health Sciences, University of Southampton, United Kingdom [2]Countess Mountbatten Hospice, Southampton, United Kingdom [3]Southampton General Hospital, Southampton, United Kingdom [4]The Rowans Hospice, Waterlooville, United Kingdom [5]Australian Institute of Health Service Management, University of Tasmania, Australia [6]Carer representative, University of Southampton, United Kingdom [7]School of Allied Health, University of Limerick, Ireland [8]University of Sheffield, United Kingdom [9]Faculty of Medicine, University of Southampton, United Kingdom [10]NIHR Evaluation, Trials and Studies Coordinating Centre (NETSCC), University of Southampton, United Kingdom [11]Motor Neurone Disease Association, Northampton, United Kingdom [12]Sheffield Teaching Hospitals NHS Foundation Trust, Sheffield, United Kingdom [13]Patient representative, University of Southampton, United Kingdom

For peer review only

ABSTRACT

Introduction:

Motor neurone disease (MND) is a progressive, incurable disease, characterised by degeneration of the nerves in the brain and spinal cord. Due to the multisystem effects of the disease, patients are faced with many complex, time sensitive decisions, one of which is the decision on gastrostomy feeding. There are currently no published decision aids to support patients making this decision in the UK. This study will develop and pilot a patient decision aid to: provide evidence-based information on gastrostomy placement and feeding that is relevant to people with MND; communicate the risks and benefits associated with each option; check understanding; and clarify personal values and preferences, enabling patients to make a decision congruent with their values and appropriate for them.

Methods and analysis:

A two phase process, observing the International Patient Decision Aid Standards (IPDAS), will be used to develop the decision aid, over 24 months starting January 2019. Phase 1 will use literature reviews and stakeholder interviews and surveys to identify essential content for the decision aid, and explore the best way to present this. In the second phase, a prototype decision aid will be developed and revised using stakeholder feedback in an iterative process. Stakeholders will include individuals with MND, their carers and the healthcare professionals (HCPs) working with them.

Ethics and dissemination:

Ethical approval for the study has been granted by West of Scotland Research Ethics Service, reference 19/WS/0078. Study findings will be disseminated through academic and non-academic publications, conference presentations, stakeholder websites and social media. A feasibility study will follow, to explore the acceptability and practicality of the decision aid for patients, carers and

HCPs in practice and to assess whether the decision aid shows promise of being beneficial for the intended population.

Keywords: motor neuron disease, gastrostomy, enteral nutrition, decision making, decision support techniques

Strengths and limitations of this study

Review of the literature will ensure all relevant evidence is captured.

A user-centric approach to development will result in a decision aid that addresses the needs of patients with motor neurone disease (MND).

Using an iterative process to determine essential content for the decision aid and to develop the prototype may be time consuming.

Allowing patients the option to have a family member, friend or carer present during the interviews has the potential to influence the honesty and openness of their responses.

INTRODUCTION

Motor neurone disease (MND) is a progressive, incurable disease, characterised by degeneration of the nerves in the brain and spinal cord, leading to muscle weakness. It is estimated that up to 5,000 people are living with MND at any one time in the UK, with reported incidence rates of 1.06 to 2.4 per 100,000 people per year(1). Typical life expectancy from symptom onset is 3 years(2). However, due to the heterogeneity of MND, prognosis is unpredictable, with some individuals living for many years after diagnosis and others far less(3). Patients will experience volatile multi-system physical deterioration over the course of their disease, which may be accompanied by cognitive and behavioural change(4), requiring them to navigate a challenging care pathway with numerous complex decisions.

Dysphagia (difficulty swallowing) is common in people with MND. Those with bulbar subtypes of the disease will typically experience dysphagia early on in their disease trajectory, with more than two-thirds of all people with MND affected in late stages of the disease(5). Dysphagia may cause distressing coughing and choking, laboured mealtimes, and aspiration, which in turn can lead to recurrent chest infections, nutritional decline and weight loss(6).

When an individual with MND presents with the signs and symptoms of dysphagia, gastrostomy tube feeding is often recommended, based on the assumption that there will be a beneficial impact on nutritional outcome, survival and quality of life (QOL)(7, 8). Gastrostomy refers to the opening made in the abdomen, through which the feeding tube enters the stomach, and as with any surgical procedure, carries a level of risk. However, there are no randomised controlled trials (RCTs) that explore the efficacy of tube feeding in MND and results from other research is equivocal, weak or lacking with respect to survival, nutritional outcome and QOL respectively(9).

Gastrostomy feeding can have a significant impact on an individual’s everyday life, with both positive and negative consequences(10). Negative impacts include difficulties with feed administration, handling the equipment and managing supplies and storage, along with potential clinical

For peer review only

complications and the psychological impact, such as anxiety around the tube and social isolation through loss of shared mealtimes. Potential benefits may include relief of anxiety associated with prolonged, effortful mealtimes by removing the need to eat and drink, weight stabilisation and perceived survival benefit. From the patient and carer perspective, this means the decision regarding whether to have a gastrostomy is not straightforward. To add to the complexity, there is also a time sensitivity to this decision, which was explored in ProGas(11), a large longitudinal prospective cohort study that investigated optimum timing and method of gastrostomy for people with MND. The study found that while earlier gastrostomy may improve survival time and nutritional outcomes, some patients might delay placement or not want a tube placing at all. This is reflected in current National Institute for Health and Care Excellence (NICE) guidelines for MND(12), which state that the role of gastrostomy feeding should be discussed at an early stage but that it must be recognised that not all individuals will wish to proceed.

Whilst there are some excellent educational resources about gastrostomy placement and feeding, including ‘myTube’, informed by the findings of ProGas(11) and online information provided by the MND Association(13), there are no published patient decision aids (DAs) for this group in the UK. DAs are designed to do more than provide information; they are usually developed for complex, preference based decisions that require greater consideration(14). These decisions will have multiple options with features or outcomes that people value differently. Often the evidence-base for each option is limited and so the best choice depends on the personal importance the individual places on the benefits, harms, burden and scientific uncertainties(14). By presenting evidence-based information, communicating the risks and benefits associated with each option, checking understanding and clarifying personal values and preferences, a DA may help individuals with MND make a decision about whether a gastrostomy is congruent with their values and appropriate for them(15). They can also provide an outline for conversations with family members and healthcare professionals, and encourage a collaborative approach to decision-making(16).

It is crucial to acknowledge the importance of involving close family members and carers in the gastrostomy decision-making process. Research has shown that carers may directly or indirectly influence patient decision-making and that patients often want to share the burden of decision-making with family and carers(17), in some cases relying on the carer or family member in order to participate in the decision-making process(18). For individuals with MND, communication difficulties secondary to their disease may augment their reliance on carers or family members. Whilst the values and preferences of the patient must take priority, developing a DA that includes content for carers ensures that these individuals are well informed and able to participate fully in discussions, thus offering the patient better support in the decision-making process. It also provides support for the carer themselves; allowing them to prepare for the potential impact of the patient’s decision on their own lives and providing sign posting to further support and information.

The development of a DA to support shared decision-making aligns with current NHS priorities of patient centred, value-based healthcare. Shared decision-making is a key component of NHS England’s personalised care approach, laid out in their Universal personalised care model(19, 20), as part of the 10 Year Plan(21). The model is underpinned by evidence that shared decision-making leads to more realistic expectations, a better match between individuals’ values and treatment choices, and fewer unnecessary interventions(20). NHS Rightcare’s value-based initiative(22) also highlights the importance of listening to patients to identify what is of value to them to ensure that their care ‘delivers’ on an individual level, and using this to design and deliver services accordingly. The DA will support these NHS approaches by empowering the patient to participate in shared decision-making regarding gastrostomy placement.

For peer review only

METHODS AND ANALYSIS

This research protocol details the process for the development and piloting of a decision aid (DA) for individuals with motor neurone disease (MND), deciding whether to have a gastrostomy tube for feeding. The study will be conducted over 24 months and started in January 2019.

The study will have two phases:

Phase 1: to determine what information should be included in the DA and the best way to present this

Phase 2: development and pilot testing of the prototype DA

This will be followed by a multi-centre feasibility study, details of which will be presented in a later publication.

Participants

Participants will include individuals from the three key stakeholder groups involved in the decision-making process: individuals living with MND (patients), their carers and the healthcare professionals working with them.

Purposive sampling(23) will be employed to optimise stakeholder representation, ensuring the decision aid (DA) content meets the needs of the UK MND population. The sampling frame will be based on time since diagnosis and gastrostomy placement (Table 1). Those participants in the 'no gastrostomy' group will include patients who have declined gastrostomy. A similar number of participants will be sought in each of the green cells. Although recruitment of patients and carers in the amber cell will be attempted, it is anticipated that the number of patients and carers that fall into this group will be small.

Table 1: Patient and carer sampling frame

	<6 months since diagnosis	>6 months since diagnosis
Gastrostomy	Aim to recruit where possible	Interview: patients n= 10, carers n= 5<br>Beta-testing: patients n= 3-4, carers n= 3-4
No gastrostomy	Interview: patients n= 10, carers n= 5<br>Beta-testing: patients n=3-4, carers n= 3-4	Interview: patients n= 10, carers n= 5<br>Beta-testing: patients n=3-4, carers n= 3-4

We aim to recruit patients who are broadly representative of the MND population in terms of age, gender and subtype of MND and we will include both people who currently live alone and those who live with others. Health care professionals will be drawn from a variety of professions including doctors, nurses and allied health professionals.

An inclusion and exclusion criteria will be applied to potential participants (Table 2). Individuals assessed as lacking capacity and their carers will be excluded because their decision-making informational needs are likely to differ significantly from those with capacity. Future research should investigate the needs of individuals with MND lacking capacity, and their care network, in gastrostomy decision making.

Table 2: Inclusion and exclusion criteria

**Inclusion criteria**	**Exclusion criteria**
Individuals with a confirmed diagnosis of motor neurone disease (MND) with a life expectancy of at least a year as reported by the clinical team	Unable to participate in English<br><br><18 years

For peer review only

OR<br>Carers, defined as 'Any adult who looks after a family member, partner or friend with motor neurone disease. The care they give is unpaid, and they have face-to-face contact with the person with MND at least 3 times a week' (currently or within the last year). They may or may not be in receipt of carers allowance.<br>OR<br>Healthcare professionals working with individuals with MND, involved in the gastrostomy decision making process or supporting patients with gastrostomy feeding<br>AND<br>Able to communicate, either verbally or using other means e.g. using a communication aid, at phrase or sentence level	Individuals assessed as lacking capacity by the clinical team<br><br>Carers of individuals assessed as lacking capacity

Design

The methods for the two phases of the study are based on a validated model for web-based decision aids(24) and the International Patient Decision Aid Standards(25), and will observe the Medical Research Council's guidance for the development of complex interventions(26). Phase 1 of the study will elicit the information that should be included in the decision aid (DA) and identify the best way to present it. In the Phase 2, the DA will be developed and revised. A study advisory committee (SAC) including individuals living with MND, their carers, and healthcare professionals and researchers with experience in MND and/or patient decision-making has been assembled and has contributed to the study design.

Phase 1: Content and Presentation

Literature reviews

A literature review to identify qualitative studies that explore the informational needs of people with neurodegenerative disease considering a gastrostomy will be conducted using standard methods(27). The search will be limited to decision making around gastrostomy, rather than including all healthcare decisions for this group, primarily because of the invasive nature of the procedure. The search will also be limited to studies of patients with neurodegenerative disease, rather than any condition where tube feeding may be offered, because of the parallels with MND experience and disease outcomes. Thematic analysis will be used to identify the information that is most valued when decision-making.

A second literature review will identify the best methods for presenting information within the context of a web-based DA. This review will search for web-based DAs and studies describing their development and evaluation, both for patients with MND and other patient groups. Methods for presenting information will be tabulated, along with any information about efficacy. Information about the theoretical underpinning of DAs will also be collated to identify the active ingredients of the decision-making process.

Interviews

Cross-sectional, semi-structured in-depth interviews with patient, carer and healthcare professional participants, will explore relevant personal experiences (individuals and carers only); important information to include when making a decision about whether to have a gastrostomy; what a DA might look like and when and how a DA should be introduced. The informational needs of

For peer review only

carers supporting individuals to make this decision will also be investigated. An interview guide, developed with support from the SAC, will be used to guide but not constrain the interviews. Interviews will be carried out either in person or by telephone, at a time and place convenient to the participant. Interviews will be audio recorded and transcribed verbatim. It is recognised that a significant proportion of patient participants will have communication difficulties. To minimise the burden of the interviews for patients, they will be sent the interview guide ahead of the interview, to allow them to begin to construct their answers. Patients will also be given the option to communicate verbally, via a communication aid, or in writing (e.g. in person or via email), if this is the only viable option. Patient and carer interviews will be conducted independently, although patient participants will be able to have a family member, friend or carer present in the interview if preferred.

Clinical (patients only) and demographic data will also be collected, during or prior to the interview, to allow description of the study sample and evaluation of whether it is representative of a typical MND population in the UK. Patient data will be obtained from medical notes, while demographic data from carers and healthcare professionals will be reported by the participant. This will include:

i. Patients: age, gender, ethnicity, level of education, employment status, domestic status, family history of MND, date of diagnosis, type of MND, site of onset, stage of disease, current functional status (ALS Functional Rating Scale), gastrostomy placed (yes or no, date if applicable and why)

ii. Carers: age, gender, ethnicity, level of education, employment status, family history of MND

iii. Healthcare professionals: years qualified, job title, time working in the field of MND.

Decision Aid Content

Interview and literature review outputs will be synthesised using a framework analysis approach(28). This information will then be prioritised by interview participants, as well as members of the SAC and study team, using the prioritisation model MoSCoW(29). For each suggested piece of content, respondents will be asked to rate how crucial it is for this to be included in the DA (M– Must have; S – Should have; C – Could have; W– Would like if time permits). Respondents will choose whether to complete the survey online, on a hard copy or via a phone interview.

Finally, a systematic search of the evidence base relating to each suggested piece of content will be carried out to ensure all information included in the DA is accurate. The search will include published papers, guidelines, best practice documents, other grey literature and input from clinical experts on the SAC.

Phase 2: Prototype development and piloting

Results from Phase 1 will inform the development of the prototype DA. Using an iterative process, and observing the IPDAS checklist, the SAC and research team will develop a prototype DA. The DA will be web-based, programmed using responsive web design, enabling use on tablets, laptops and smart phones. The prototype will then undergo alpha testing; where phase 1 study participants will review the prototype and complete a survey to provide feedback on:

1. Clarity of prototype information
2. Presentation and functionality of prototype information (user friendliness and acceptability)
3. Perceptions of utility

It is recognised that between the interview and alpha testing (expected timeframe of 3-8 months depending on when the participant is recruited), a proportion of patient participants may become too unwell to participate, or will die. The period between interview and alpha testing will be kept as short as possible, however if a patient is unable to participate in the alpha testing this will be treated as missing data.

For peer review only

Feedback will be discussed with the SAC and a second version of the prototype DA will then be produced. Version 2 will undergo beta-testing; newly recruited patients, carers and healthcare professionals (n=5 of each) will talk through their use of the DA following the "think aloud" method(30). A think aloud interview guide will be used to prompt participants to verbalise their thoughts as they use the DA, followed by a number of evaluation questions immediately after completion. The SAC and study team will then meet again to review the results and produce version 3 of the prototype, which will undergo beta-testing with a new set of participants (n=5 of each group). Finally, the SAC and study team will decide whether any additional changes are required and the final prototype will be produced.

Recruitment

Patient and carer participants will be identified by a member of their clinical care team in NHS MND and palliative care clinics across the Wessex and Sheffield regions. Permission will be obtained to share the contact details of any individual interested in participating, with the research team. Support groups, such as those run by the Motor Neurone Disease Association, will provide another avenue for the recruitment of patients and carers. A member of the research team will attend the local support group in person to introduce the study, provide a participant information sheet to those interested in participating and request permission to make follow up contact. A third channel for patient and carer recruitment will be advertisements placed on relevant websites and social media platforms e.g. Twitter. Interested individuals will be invited to contact the researcher, who will provide a participant information sheet and request permission to make follow up contact. The clinical care team will be consulted to confirm the eligibility of all patient participants.

Eligible healthcare professionals will be identified either following direct approach by the researcher, via a member of the advisory committee (who will obtain consent to share their contact details with the researcher), or through adverts via social media and professional groups. A researcher will discuss the study with the individual, provide a participant information sheet and request permission to make follow up contact.

All participants will be asked to provide informed consent prior to participation in the study.

Patient and public involvement

People living with MND and their carers will be involved throughout the research process. The idea of developing a decision aid for people with MND considering a gastrostomy was initially presented to an MND support group for feedback and to invite members to join the research team.

Following award of the grant, people living with MND and their carers have joined the SAC; through this they will help to prioritise content for the DA, evaluate each iteration of the DA and comment on all patient and carer facing study documents. They will also advise on the practicalities of including people with MND as research participants and have input on the recruitment strategy.

The views and insights of patient and carer participants will be central to the research and will form the majority of the study sample. Participants will be asked whether they wish to be kept up to date with the project's progress and those who do will be sent a newsletter.

ETHICS AND DISSEMINATION

For peer review only

Ethical approval for the study has been granted by West of Scotland Research Ethics Service, reference 19/WS/0078. Participant information sheets, consent forms, recruitment advertisements and GP letters were also approved. All amendments to study documents will be submitted in IRAS for HRA and REC approval.

Consent

The researcher will obtain written informed consent from participants prior to them entering into the study. For patient participants, this will include seeking permission to inform their GP of their participation in the study. In the case that the individual is unable to provide written consent, consent will be indicated by the participant answering 'yes' or 'no', either verbally or via a communication aid (e.g. tablet device or Lightwriter) after each statement on the consent form. The form will then be signed by the parties involved in the consent conversation(31).

Given the sensitive nature of the topics to be discussed, the researcher will make clear that participation is voluntary, and the right to ask any questions and to decline participation at any time will be highlighted during the data collection.

Participant Confidentiality

The study will adhere to the principles of Good Clinical Practice (GCP) and in accordance with current Data Protection Regulations, the Data Protection Act 2018 (GDPR) and all other regulatory requirements, as appropriate. Necessary steps will be taken to ensure all data remain confidential at all times. Study data will be kept for 10 years from the end of the study, in accordance with University of Southampton policy. Only members of the research team will have access to the full dataset.

Dissemination

A dissemination and impact plan will be developed in the initial stages of the study. Dissemination will be led by working collaboratively with Marie Curie and the Motor Neurone Disease Association (MNDA) (the Funders) and Public Policy, University of Southampton, to develop and take forward the impact activities to ensure maximum publicity and benefit. Study findings will be disseminated via multiple channels, including peer-reviewed academic publications, non-academic publications, conference presentations, stakeholder websites and social media. All participants will be notified of the outcome of the study by newsletter, press release and infographic.

Once a final prototype has been developed, a multi-centre feasibility study will be carried out to explore the acceptability and practicality of the DA for patients, carers and HCPs in practice, and to assess whether the DA shows promise of being beneficial for the intended population. The protocol for the feasibility study will be presented in a subsequent publication. On completion of the feasibility study, it is anticipated that there will be a DA that can be used in clinical practice with the aim of improving the decision-making process and quality of decision for individuals with MND deciding whether to have a gastrostomy. It is also hoped that relevant national MND guidelines will make reference to the tool. The project therefore has the potential for multifaceted impact by advancing patient care, improving service delivery and providing useful insights for the development of decision aids in the future.

Twitter @DiAMoNDstudy_

Contributors RE and SW led on the design of the study and prepared the manuscript, with contributions from all authors.

Acknowledgements CJ McDermott is supported by the Sheffield NIHR BRC.

For peer review only - http://bmjopen.bmj.com/site/about/guidelines.xhtml

Funding This work was supported by Marie Curie and the Motor Neurone Disease Association grant numbers: MCRGS-20171219-8005 and 963-794 respectively.

Competing interests None declared.

Data statement Data available on request from the corresponding author.

REFERENCES

1 Hoppitt T, Pall H, Calvert M, et al. A systematic review of the incidence and prevalence of long-term neurological conditions in the UK. *Neuroepidemiology* 2011;36(1):19-28.
2 Hardiman O, van den Berg LH, Kiernan MC. Clinical diagnosis and management of amyotrophic lateral sclerosis. *Nat Rev Neurol* 2011;7(11):639-49.
3 Motor Neurone Disease Association. What is MND? 2018 [Available from: https://www.mndassociation.org/about-mnd/where-do-i-start/what-is-mnd/ Access date: June 2019].
4 Baumer D, Talbot K, Turner MR. Advances in motor neurone disease. *J R Soc Med* 2014;107(1):14-21.
5 Motor Neurone Disease Association. Dysphagia [Available from: https://www.mndassociation.org/forprofessionals/mndmanagement/dysphagia/ Access date: June 2019]
6 O'Rourke F, Vickers K, Upton C, et al. Swallowing and oropharyngeal dysphagia. *Clin Med* 2014;14(2):196-9.
7 Andersen PM, Borasio GD, Dengler R, et al. EFNS task force on management of amyotrophic lateral sclerosis: guidelines for diagnosing and clinical care of patients and relatives. *Eur J Neurol* 2005;12(12):921-38.
8 Miller RG, Jackson CE, Kasarskis EJ, et al. Practice parameter update: the care of the patient with amyotrophic lateral sclerosis: drug, nutritional, and respiratory therapies (an evidence-based review): report of the Quality Standards Subcommittee of the American Academy of Neurology. *Neurology* 2009;73(15):1218-26.
9 Katzberg HD, Benatar M. Enteral tube feeding for amyotrophic lateral sclerosis/motor neuron disease. *Cochrane Database of Systematic Reviews* 2011(1):CD004030.
10 Stavroulakis T, Baird WO, Baxter SK, et al. The impact of gastrostomy in motor neurone disease: challenges and benefits from a patient and carer perspective. *BMJ support* 2016;6(1):52-9.
11 ProGas Study Group. Gastrostomy in patients with amyotrophic lateral sclerosis (ProGas): a prospective cohort study. *Lancet Neurol* 2015;14(7):702-9.
12 National Institute for Health and Care Excellence (NICE). Motor neurone disease: assessment and management 2016 [Available from: https://www.nice.org.uk/guidance/ng42/chapter/recommendations#nutrition-and-gastrostomy Access date: June 2019]
13 Motor Neurone Disease Association. Living with MND: Swallowing, eating and drinking. 2019 [Available from: https://www.mndassociation.org/about-mnd/living-with-mnd/eating-and-drinking/ Access date: June 2019].
14 International Patient Decision Aid Standards (IPDAS) Collaboration. What are patient decision aids 2017 [Available from: http://ipdas.ohri.ca/what.html Access date: June 2019].
15 Hogden A, Greenfield D, Caga J, et al. Development of patient decision support tools for motor neuron disease using stakeholder consultation: a study protocol. *BMJ Open* 2016;6(4).
16 Alden DL, Friend J, Schapira M, et al. Cultural targeting and tailoring of shared decision making technology: a theoretical framework for improving the effectiveness of patient decision aids in culturally diverse groups. *Soc Sci Med* 2014;105:1-8.

For peer review only

17 Greenaway LP, Martin NH, Lawrence V. Accepting or declining non-invasive ventilation or gastrostomy in amyotrophic lateral sclerosis: patients' perspectives. *Journal of Neurology* 2015;262:1002-13.
18 Morris MA, Dudgeon BJ, Yorkston K. A qualitative study of adult AAC users' experiences communicating with medical providers. *Disabil Rehabil Assist Technol* 2013;8:472-81.
19 NHS England. Shared Decision Making [Available from: https://www.england.nhs.uk/shared-decision-making/ Access date: June 2019]
20 NHS England. Universal personalised care [Available from: https://www.england.nhs.uk/personalisedcare/upc/comprehensive-model/ Access date: June 2019]
21 NHS. The NHS Long Term Plan. 2019 [Available from: www.longtermplan.nhs.uk Access date: June 2019]
22 NHS Confederation. Taking the value-based agenda forward: The five essential components of value-based approaches to health and care. 2018 [Available from: https://www.nhsconfed.org/resources/2018/09/taking-the-value-based-agenda-forward Access date: June 2019]
23 Patton MQ. Qualitative Research and Evaluation Methods. Thousand Oaks, CA: Sage Publications; 2002.
24 Elwyn G, Kreuwel I, Durand MA, et al. How to develop web-based decision support interventions for patients: a process map. *Patient Educ Couns* 2011;82(2):260-5.
25 Elwyn G, et al on behalf of the International Patient Decision Aids Standards (IPDAS) Collaboration. Developing a quality criteria framework for patient decision aids: online international Delphi consensus process. *BMJ* 2006;333:417.
26 Medical Research Council. Developing and evaluating complex interventions: new guidance. *BMJ* 2008;337.
27 Centre for Reviews and Dissemination. Systematic reviews: CRD's guidance for undertaking reviews in health care 2009 [Available from: https://www.york.ac.uk/media/crd/Systematic_Reviews.pdf Access date: June 2019]
28 Gale NK, Heath G, Cameron E, et al. Using the framework method for the analysis of qualitative data in multidisciplinary health research. *BMC Med Res Methodol* 2013;13:117.
29 DSDM Agile Project Framework Handbook. MoSCoW Prioritisation. [Available from: https://www.agilebusiness.org/content/moscow-prioritisation Access date: June 2019]
30 Yardley L, Morrison LG, Andreou P, et al. Understanding reactions to an internet-delivered health-care intervention: accommodating user preferences for information provision. *BMC Med Inform Decis Mak* 2010;10(1):52.
31 NHS Health Research Authority. Consent and Participant Information Guidance: Principles of consent: General principles and role of Participant Information Sheets [Available from: http://www.hra-decisiontools.org.uk/consent/principles-general.html Access date: June 2019]

For peer review only

BMJ Open

DEVELOPING A WEB-BASED PATIENT DECISION AID FOR GASTROSTOMY IN MOTOR NEURONE DISEASE: A STUDY PROTOCOL

Journal:	*BMJ Open*
Manuscript ID	bmjopen-2019-032364.R1
Article Type:	Protocol
Date Submitted by the Author:	01-Oct-2019
Complete List of Authors:	Maunsell, Rose; University of Southampton, Health Sciences<br>Bloomfield, Suzanne; Countess Mountbatten House<br>Erridge, Clare; Southampton General Hospital<br>Foster, Claire; University of Southampton, Health Sciences<br>Hardcastle, Maggi; Rowans Hospice<br>Hogden, Anne; University of Tasmania, Australian Institute of Health Service Management<br>Kidd, Alison; University of Southampton, Carer representative<br>Lisiecka, Dominika; University of Limerick, School of Allied Health<br>McDermott, Christopher; university of sheffield,<br>Morrison, Karen; University of Southampton, Faculty of Medicine<br>Recio-Saucedo, Alejandra; University of Southampton<br>Rickenbach, Louise; Motor Neurone Disease Association<br>White, Sean; Sheffield Teaching Hospitals NHS Foundation Trust, Dietetic Department<br>Williams, Peter; University of Southampton, Patient representative<br>Wheelwright, Sally J.; Univ Southampton, Health Sciences
**Primary Subject Heading**:	Neurology
Secondary Subject Heading:	Nutrition and metabolism, Qualitative research, Research methods
Keywords:	Motor neurone disease < NEUROLOGY, NUTRITION & DIETETICS, Adult palliative care < PALLIATIVE CARE, Protocols & guidelines < HEALTH SERVICES ADMINISTRATION & MANAGEMENT

SCHOLARONE™
Manuscripts

DEVELOPING A WEB-BASED PATIENT DECISION AID FOR GASTROSTOMY IN MOTOR NEURONE DISEASE: A STUDY PROTOCOL

Corresponding Author

Dr Sally Wheelwright
Health Sciences,
University Of Southampton,
SO17 1BJ
Email: S.J.Wheelwright@soton.ac.uk
Tel: 02380 597860

Co-authors

Mrs Rose Maunsell
Health Sciences,
University Of Southampton,
SO17 1BJ
Email: r.evill@soton.ac.uk
Tel: 02380 597939

Ms Suzanne Bloomfield
Community CNS in Palliative Care Team
Countess Mountbatten Hospice
Southampton, SO30 3JB
Tel: 02380 475874
Email: suzanne.bloomfield@uhs.nhs.uk

Ms Clare Erridge
Lead Motor Neurone Disease Care Centre Coordinator Mailpoint 101 Wessex Neurological Centre
Southampton General Hospital
Tremona Road
Southampton, SO16 6YD
Tel: 02381 206544
Email: clare.erridge@uhs.nhs.uk

Professor Claire Foster
Professor of Psychosocial Oncology
School of Health Sciences
University of Southampton
SO17 1 BJ
Tel: (023) 8059 4006
Email: C.L.Foster@soton.ac.uk

Ms Maggi Hardcastle
Clinical Specialist Physiotherapist
The Rowans Hospice
Purbrook Heath Road
Waterlooville, PO7 5RU
Tel: 02392 250001
Email: maggi.hardcastle@rowanshospice.co.uk

Dr Anne Hogden
Senior Lecturer

For peer review only

Australian Institute of Health Service Management
University of Tasmania, Rozelle Campus
Cnr Glover and Church Streets, Lilyfield, NSW 2040
Tel: +61 2 8572 7922
Email: anne.hogden@utas.edu.au

Dr Alison Kidd
Lay person
Corlan, Pencelli, Brecon, Powys, LD3 7LX
Tel: 091874 665401
Email: alison@prospectory.co.uk

Dr Dominika Lisiecka
Lecturer in Speech and Language Therapy
School of Allied Health
Health Sciences Building
Room H2-021
University of Limerick
Email: dominika.lisiecka@ul.ie

Professor Christopher McDermott
Professor of Translational Neurology / Consultant Neurologist
Sheffield Institute for Translational Neuroscience
385a Glossop Rd
Sheffield, S10 2HQ
Tel: 0114 2222295 (Secretary)
Email: c.j.mcdermott@sheffield.ac.uk

Professor Karen Morrison
Associate Dean for Education and Professor of Neurology
Faculty of Medicine, University of Southampton
Room AB217, Level B, South Academic Block, MP801, Southampton General Hospital, SO16 6YD
Tel: 02381 208134
Email: K.E.Morrison@soton.ac.uk

Dr Alejandra Recio-Saucedo
Research Fellow, Portfolio Insight and Publications
NIHR Evaluation, Trials and Studies Coordinating Centre (NETSCC)
University of Southampton
Alpha House
Enterprise Road
Southampton, SO16 7NS
Tel: 02380 595606
Email: ars@ecs.soton.ac.uk

Ms Louise Rickenbach
Regional Care Development Adviser for South Central
Motor Neurone Disease Association
Tel: 03453751831
Email: louise.rickenbach@mndassociation.org

Mr Sean White
Dietetic Department
Nurses Home
Northern General Hospital

For peer review only

Herries Road
Sheffield S5 7AU
Tel: 0114 2714162
E-mail: sean.white@sth.nhs.uk

Mr Peter Williams
Lay Person
Corlan, Pencelli, Brecon, Powys, LD3 7LX
Tel: 091874 665401
Email: peter@prospectory.co.uk

Dr Sally Wheelwright
Senior Research Fellow
School of Health Sciences
University of Southampton
SO17 1BJ
Tel: (023) 8059 7860
Email: S.J.Wheelwright@soton.ac.uk

For peer review only

Word count, excluding title page, abstract, references, figures and tables: 3832

DEVELOPING A WEB-BASED PATIENT DECISION AID FOR GASTROSTOMY IN MOTOR NEURONE DISEASE: A STUDY PROTOCOL

Rose Maunsell[1], Suzanne Bloomfield[2], Clare Erridge[3], Claire Foster[1], Maggi Hardcastle[4], Anne Hogden[5], Alison Kidd[6], Dominika Lisiecka[7], Christopher J McDermott[8], Karen Morrison[9], Alejandra Recio-Saucedo[10], Louise Rickenbach[11], Sean White[12], Peter Williams[13] and Sally Wheelwright[1]

[1]Health Sciences, University of Southampton, United Kingdom [2]Countess Mountbatten Hospice, Southampton, United Kingdom [3]Southampton General Hospital, Southampton, United Kingdom [4]The Rowans Hospice, Waterlooville, United Kingdom [5]Australian Institute of Health Service Management, University of Tasmania, Australia [6]Carer representative, University of Southampton, United Kingdom [7]School of Allied Health, University of Limerick, Ireland [8]University of Sheffield, United Kingdom [9]Faculty of Medicine, University of Southampton, United Kingdom [10]NIHR Evaluation, Trials and Studies Coordinating Centre (NETSCC), University of Southampton, United Kingdom [11]Motor Neurone Disease Association, Northampton, United Kingdom [12]Sheffield Teaching Hospitals NHS Foundation Trust, Sheffield, United Kingdom [13]Patient representative, University of Southampton, United Kingdom

ABSTRACT

Introduction:

Motor neurone disease (MND) is a progressive, incurable disease, characterised by degeneration of the nerves in the brain and spinal cord. Due to the multisystem effects of the disease, patients are faced with many complex, time sensitive decisions, one of which is the decision on gastrostomy feeding. There are currently no published decision aids to support patients making this decision in the UK. This study will develop and pilot a patient decision aid to: provide evidence-based information on gastrostomy placement and feeding that is relevant to people with MND; communicate the risks and benefits associated with each option; check understanding; and clarify personal values and preferences, enabling patients to make a decision congruent with their values and appropriate for them.

Methods and analysis:

A two phase process, observing the International Patient Decision Aid Standards (IPDAS), will be used to develop the decision aid, over 24 months starting January 2019. Phase 1 will use literature reviews and stakeholder interviews and surveys to identify essential content for the decision aid, and explore the best way to present this. In the second phase, a prototype decision aid will be developed and revised using stakeholder feedback in an iterative process. Stakeholders will include individuals with MND, their carers and the healthcare professionals (HCPs) working with them.

Ethics and dissemination:

Ethical approval for the study has been granted by West of Scotland Research Ethics Service, reference 19/WS/0078. Study findings will be disseminated through academic and non-academic publications, conference presentations, stakeholder websites and social media. A feasibility study will follow, to explore the acceptability and practicality of the decision aid for patients, carers and HCPs in practice and to assess whether the decision aid shows promise of being beneficial for the intended population.

Keywords: motor neuron disease, gastrostomy, enteral nutrition, decision making, decision support techniques

Strengths and limitations of this study

Review of the literature will ensure all relevant evidence is captured.

A user-centric approach to development will result in a decision aid that addresses the needs of patients with motor neurone disease (MND).

Using an iterative process to determine essential content for the decision aid and to develop the prototype may be time consuming.

Allowing patients the option to have a family member, friend or carer present during the interviews has the potential to influence the honesty and openness of their responses.

INTRODUCTION

Motor neurone disease (MND) is a progressive, incurable disease, characterised by degeneration of the nerves in the brain and spinal cord, leading to muscle weakness. It is estimated that up to 5,000 people are living with MND at any one time in the UK, with reported incidence rates of 1.06 to 2.4 per 100,000 people per year(1). Typical life expectancy from symptom onset is 3 years(2). However, due to the heterogeneity of MND, prognosis is unpredictable, with some individuals living for many years after diagnosis and others far less(3). Patients will experience volatile multi-system physical deterioration over the course of their disease, which may be accompanied by cognitive and behavioural change(4), requiring them to navigate a challenging care pathway with numerous complex decisions.

Dysphagia (difficulty swallowing) is common in people with MND. Those with bulbar subtypes of the disease will typically experience dysphagia early on in their disease trajectory, with more than two-thirds of all people with MND affected in late stages of the disease(5). Dysphagia may cause

distressing coughing and choking, laboured mealtimes, and aspiration, which in turn can lead to recurrent chest infections, nutritional decline and weight loss(6).

When an individual with MND presents with the signs and symptoms of dysphagia, gastrostomy tube feeding is often recommended, based on the assumption that there will be a beneficial impact on nutritional outcome, survival and quality of life (QOL)(7, 8). Gastrostomy refers to the opening made in the abdomen, through which the feeding tube enters the stomach, and as with any surgical procedure, carries a level of risk. However, there are no randomised controlled trials (RCTs) that explore the efficacy of tube feeding in MND and results from other research is equivocal, weak or lacking with respect to survival, nutritional outcome and QOL respectively(9).

Gastrostomy feeding can have a significant impact on an individual's everyday life, with both positive and negative consequences(10). Negative impacts include difficulties with feed administration, handling the equipment and managing supplies and storage, along with potential clinical complications and the psychological impact, such as anxiety around the tube and social isolation through loss of shared mealtimes. Potential benefits may include relief of anxiety associated with prolonged, effortful mealtimes by removing the need to eat and drink, weight stabilisation and perceived survival benefit. From the patient and carer perspective, this means the decision regarding whether to have a gastrostomy is not straightforward. To add to the complexity, there is also a time sensitivity to this decision, which was explored in ProGas(11), a large longitudinal prospective cohort study that investigated optimum timing and method of gastrostomy for people with MND. The study found that while earlier gastrostomy may improve survival time and nutritional outcomes, some patients might delay placement or not want a tube placing at all. This is reflected in current National Institute for Health and Care Excellence (NICE) guidelines for MND(12), which state that the role of gastrostomy feeding should be discussed at an early stage but that it must be recognised that not all individuals will wish to proceed.

Whilst there are some excellent educational resources about gastrostomy placement and feeding, including 'myTube', informed by the findings of ProGas(11) and online information provided by the MND Association(13), there are no published patient decision aids (DAs) for this group in the UK. DAs are designed to do more than provide information; they are usually developed for complex, preference based decisions that require greater consideration(14). These decisions will often have multiple options with features or outcomes that people value differently, in this case: undergoing gastrostomy placement, deciding against gastrostomy, or deciding to revisit the issue at a later date. Often the evidence-base for each option is limited and so the best choice depends on the personal importance the individual places on the benefits, harms, burden and scientific uncertainties(14). By presenting evidence-based information, communicating the risks and benefits associated with each option, checking understanding and clarifying personal values and preferences, a DA may help individuals with MND make a decision about whether a gastrostomy is congruent with their values and appropriate for them(15). They can also provide an outline for conversations with family members and healthcare professionals, and encourage a collaborative approach to decision-making(16). It is crucial to acknowledge the importance of involving close family members and carers in the gastrostomy decision-making process. Research has shown that carers may directly or indirectly influence patient decision-making and that patients often want to share the burden of decision-making with family and carers(17), in some cases relying on the carer or family member in order to participate in the decision-making process(18). For individuals with MND, communication difficulties secondary to their disease may augment their reliance on carers or family members. Whilst the values and preferences of the patient must take priority, developing a DA that includes content for carers ensures that these individuals are well informed and able to participate fully in

For peer review only

discussions, thus offering the patient better support in the decision-making process. It also provides support for the carer themselves; allowing them to prepare for the potential impact of the patient's decision on their own lives and providing sign posting to further support and information.

The development of a DA to support shared decision-making aligns with current NHS priorities of patient centred, value-based healthcare. Shared decision-making is a key component of NHS England's personalised care approach, laid out in their Universal personalised care model(19, 20), as part of the 10 Year Plan(21). The model is underpinned by evidence that shared decision-making leads to more realistic expectations, a better match between individuals' values and treatment choices, and fewer unnecessary interventions(20). NHS Rightcare's value-based initiative(22) also highlights the importance of listening to patients to identify what is of value to them to ensure that their care 'delivers' on an individual level, and using this to design and deliver services accordingly. The DA will support these NHS approaches by empowering the patient to participate in shared decision-making regarding gastrostomy placement.

The study will answer the following research questions:

1. What information should be included in a DA for people with MND considering whether to have a gastrostomy, their carers and health care professionals, and how is this best presented?

2. Do people with MND, carers and health care professionals find a web-based DA easy to use and useful?

METHODS AND ANALYSIS

This research protocol details the process for the development and piloting of a decision aid (DA) for individuals with motor neurone disease (MND), deciding whether to have a gastrostomy tube for feeding. The study will be conducted over 24 months and started in January 2019.

The study will have two phases:

Phase 1: will address the first research question by investigating what information should be included in the DA and how this is best presented.

Phase 2: the prototype DA will be developed, and the second research question will be addressed by exploring whether the prototype DA is easy to use and useful to the end user/s.

This will be followed by a multi-centre feasibility study (Phase 3) to explore the acceptability and practicality of the DA for patients, carers and HCPs in practice, and to assess whether the DA shows promise of being beneficial for the intended population. Details of which will be presented in a later publication.

Participants

Participants will include individuals from the three key stakeholder groups involved in the decision-making process: individuals living with MND (patients), their carers and the healthcare professionals working with them.

Purposive sampling(23) will be employed to optimise stakeholder representation, ensuring the decision aid (DA) content meets the needs of the UK MND population. The sampling frame will be based on time since diagnosis and gastrostomy placement (Table 1). Those participants in the 'no gastrostomy' group will include patients who have declined gastrostomy. A similar number of participants will be sought in each of the green cells. Although recruitment of patients and carers in

For peer review only - http://bmjopen.bmj.com/site/about/guidelines.xhtml

the amber cell will be attempted, it is anticipated that the number of patients and carers that fall into this group will be small.

Table 1: Patient and carer sampling frame

	<6 months since diagnosis	>6 months since diagnosis
Gastrostomy	Aim to recruit where possible	Interview: patients n= 10, carers n= 5<br>Beta-testing: patients n= 3-4, carers n= 3-4
No gastrostomy	Interview: patients n= 10, carers n= 5<br>Beta-testing: patients n=3-4, carers n= 3-4	Interview: patients n= 10, carers n= 5<br>Beta-testing: patients n=3-4, carers n= 3-4

We aim to recruit patients who are broadly representative of the MND population in terms of age, gender and subtype of MND and we will include both people who currently live alone and those who live with others. Health care professionals will be drawn from a variety of professions including doctors, nurses and allied health professionals.

An inclusion and exclusion criteria will be applied to potential participants (Table 2). The clinical care team will be consulted to confirm whether the individual has capacity to provide informed consent. Individuals assessed as lacking capacity and their carers will be excluded because their decision-making informational needs are likely to differ significantly from those with capacity. Future research should investigate the needs of individuals with MND lacking capacity, and their care network, in gastrostomy decision making.

Table 2: Inclusion and exclusion criteria

**Inclusion criteria**	**Exclusion criteria**
Individuals with a confirmed diagnosis of motor neurone disease (MND) with a life expectancy of at least a year as reported by the clinical team<br><br>OR<br>Carers, defined as 'Any adult who looks after a family member, partner or friend with motor neurone disease. The care they give is unpaid, and they have face-to-face contact with the person with MND at least 3 times a week' (currently or within the last year). They may or may not be in receipt of carers allowance.<br><br>OR<br>Healthcare professionals working with individuals with MND, involved in the gastrostomy decision making process or supporting patients with gastrostomy feeding<br><br>AND<br>Able to communicate, either verbally or using other means e.g. using a communication aid, at phrase or sentence level	Unable to participate in English<br><br><18 years<br><br>Individuals assessed as lacking capacity by the clinical team<br><br>Carers of individuals assessed as lacking capacity

Design

The methods for the two phases of the study are based on a validated model for web-based decision aids(24) and the original 2005 International Patient Decision Aid Standards(25), and will observe the Medical Research Council's guidance for the development of complex interventions(26). Phase 1 of the study will elicit the information that should be included in the decision aid (DA) and identify the best way to present it. In the Phase 2, the DA will be developed and revised. A study advisory

For peer review only

committee (SAC) including individuals living with MND, their carers, and healthcare professionals and researchers with experience in MND and/or patient decision-making has been assembled and has contributed to the study design.

Phase 1: Content and Presentation

Literature reviews
A literature review to identify qualitative studies that explore the informational needs of people with neurodegenerative disease considering a gastrostomy will be conducted using standard methods(27). The search will be limited to decision making around gastrostomy, rather than including all healthcare decisions for this group, primarily because of the invasive nature of the procedure. The search will also be limited to studies of patients with neurodegenerative disease, rather than any condition where tube feeding may be offered, because of the parallels with MND experience and disease outcomes. Thematic analysis will be used to identify information that is valued when decision-making.

A second literature review will identify theories that could be used to underpin the DA and also the best methods for presenting information within the context of a web-based DA. This review will search for web-based DAs and studies describing their development and evaluation, both for patients with MND and other patient groups. Methods for presenting information will be tabulated, along with any information about efficacy. Information about the theoretical underpinning of DAs will also be collated to help with the selection of a theory to underpin the DA and to identify the active ingredients of the decision-making process. Final theory selection will be driven by the findings from the interviews. For example, if people want to get the 'gist' in the first layer of information, then delve deeper for more detail at their discretion, the Fuzzy Trace Theory (28) may be applied. The intention is to publish both literature reviews in a separate publication(s).

Interviews
Cross-sectional, semi-structured in-depth interviews with patient, carer and healthcare professional participants, will explore relevant personal experiences (individuals and carers only); important information to include when making a decision about whether to have a gastrostomy; what a DA might look like and when and how a DA should be introduced. The informational needs of carers supporting individuals to make this decision will also be investigated. An interview guide (see Appendix 1), developed with support from the SAC, will be used to guide but not constrain the interviews. Interviews will be carried out either in person or by telephone, at a time and place convenient to the participant. Interviews will be audio recorded and transcribed verbatim. It is recognised that a significant proportion of patient participants will have communication difficulties(29). To minimise the burden of the interviews for patients, they will be sent the interview guide ahead of the interview, to allow them to begin to construct their answers. Patients may respond verbally, or by using an alternative form of communication. This may include electronic communication devices, such as a smartphone or tablet device (30), or in writing (e.g. in person or via email). Patient and carer interviews will be conducted independently, although patient participants will be able to have a family member, friend or carer present in the interview if preferred.
Clinical (patients only) and demographic data will also be collected, during or prior to the interview, to allow description of the study sample and evaluation of whether it is representative of a typical MND population in the UK. Patient data will be obtained from medical notes, while demographic data from carers and healthcare professionals will be reported by the participant. This will include:

i. Patients: age, gender, ethnicity, level of education, employment status, domestic status, family history of MND, date of diagnosis, type of MND, site of onset, stage of disease, gastrostomy placed (yes or no, date if applicable and why)

For peer review only - http://bmjopen.bmj.com/site/about/guidelines.xhtml

ii. Carers: age, gender, ethnicity, level of education, employment status, family history of MND
iii. Healthcare professionals: years qualified, job title, time working in the field of MND.

Decision Aid Content
Interview and literature review outputs will be synthesised using a framework analysis approach(31). The lead researcher will begin by identifying categories and themes in the transcripts and developing a code for each. Codes will then be discussed by the research team to ensure they are also representative of the literature review findings. Consensus will be reached through team discussion. These codes will then be applied to the whole dataset, with refinement as needed. The identified themes will be used to develop a survey to inform the selection of content for the DA. The survey will be sent to the participants who were previously interviewed, as well as members of the SAC and study team, who will all be asked to prioritise each suggested piece of content using the prioritisation model MoSCoW(32). For each piece of content, respondents will be asked to rate how crucial it is for this to be included in the DA (M– Must have; S – Should have; C – Could have; W– Would like if time permits). Respondents will choose whether to complete the survey online, on a hard copy or over the telephone.

Finally, a systematic search of the evidence base relating to each suggested piece of content will be carried out to ensure all information included in the DA is accurate. The search will include published papers, guidelines, best practice documents, other grey literature and input from clinical experts on the SAC.

Phase 2: Prototype development and piloting of the decision aid

Results from Phase 1 will inform the development of the prototype DA. The SAC and research team will use the findings from the interviews to select the relevant theory from those identified in the literature review to underpin the DA. Using an iterative process, and observing the 2005 IPDAS checklist (25), the SAC and research team will develop a prototype DA. The DA will be web-based, programmed using responsive web design, enabling use on tablets, laptops and smart phones. The prototype will then undergo alpha testing; where phase 1 study participants will review the prototype and complete a survey to provide feedback on:

1. Clarity of prototype information
2. Presentation and functionality of prototype information (user friendliness and acceptability)
3. Perceptions of utility

It is recognised that between the interview and alpha testing (expected timeframe of 3-8 months depending on when the participant is recruited), a proportion of patient participants may become too unwell to participate, or will die. The period between interview and alpha testing will be kept as short as possible, however if a patient is unable to participate in the alpha testing this will be treated as missing data.

Feedback will be discussed with the SAC and a second version of the prototype DA will then be produced. Version 2 will undergo beta-testing; newly recruited patients, carers and healthcare professionals (n=5 of each) will talk through their use of the DA following the “think aloud” method(33). A think aloud interview guide (see Appendix 2) will be used to prompt participants to verbalise their thoughts as they use the DA, followed by a number of evaluation questions immediately after completion. The SAC and study team will then meet again to review the results and produce version 3 of the prototype, which will undergo beta-testing with a new set of participants (n=5 of each group). Finally, the SAC and study team will decide whether any additional changes are required and the final prototype will be produced.

Recruitment

For peer review only

Patient and carer participants will be identified by a member of their clinical care team in NHS MND and palliative care clinics across the Wessex and Sheffield regions. Permission will be obtained to share the contact details of any individual interested in participating, with the research team. Support groups, such as those run by the Motor Neurone Disease Association, will provide another avenue for the recruitment of patients and carers. A member of the research team will attend the local support group in person to introduce the study, provide a participant information sheet to those interested in participating and request permission to make follow up contact. A third channel for patient and carer recruitment will be advertisements placed on relevant websites and social media platforms e.g. Twitter. Interested individuals will be invited to contact the researcher, who will provide a participant information sheet and request permission to make follow up contact. The clinical care team will be consulted to confirm the eligibility of all patient participants.

Eligible healthcare professionals will be identified either following direct approach by the researcher, via a member of the advisory committee (who will obtain consent to share their contact details with the researcher), or through adverts via social media and professional groups. A researcher will discuss the study with the individual, provide a participant information sheet and request permission to make follow up contact.

All participants will be asked to provide informed consent prior to participation in the study.

Patient and public involvement

People living with MND and their carers will be involved throughout the research process. The idea of developing a decision aid for people with MND considering a gastrostomy was initially presented to an MND support group for feedback and to invite members to join the research team.

Following award of the grant, people living with MND and their carers have joined the SAC; through this they will help to prioritise content for the DA, evaluate each iteration of the DA and comment on all patient and carer facing study documents. They will also advise on the practicalities of including people with MND as research participants and have input on the recruitment strategy.

The views and insights of patient and carer participants will be central to the research and will form the majority of the study sample. Participants will be asked whether they wish to be kept up to date with the project's progress and those who do will be sent a newsletter.

ETHICS AND DISSEMINATION

Ethical approval for the study has been granted by West of Scotland Research Ethics Service, reference 19/WS/0078. Participant information sheets, consent forms, recruitment advertisements and GP letters were also approved. All amendments to study documents will be submitted in IRAS for HRA and REC approval.

Consent

The researcher will obtain written informed consent from participants prior to them entering into the study. For patient participants, this will include seeking permission to inform their GP of their participation in the study. In the case that the individual is unable to provide written consent, consent will be indicated by the participant answering 'yes' or 'no', either verbally or via a communication device, after each statement on the consent form. The form will then be signed by the parties involved in the consent conversation(34).

For peer review only

Given the sensitive nature of the topics to be discussed, the researcher will make clear that participation is voluntary, and the right to ask any questions and to decline participation at any time will be highlighted during the data collection.

Participant Confidentiality

The study will adhere to the principles of Good Clinical Practice (GCP) and in accordance with current Data Protection Regulations, the Data Protection Act 2018 (GDPR) and all other regulatory requirements, as appropriate. Necessary steps will be taken to ensure all data remain confidential at all times. Study data will be kept for 10 years from the end of the study, in accordance with University of Southampton policy. Only members of the research team will have access to the full dataset.

Dissemination

A dissemination and impact plan will be developed in the initial stages of the study. Dissemination will be led by working collaboratively with Marie Curie and the Motor Neurone Disease Association (MNDA) (the Funders) and Public Policy, University of Southampton, to develop and take forward the impact activities to ensure maximum publicity and benefit. Study findings will be disseminated via multiple channels, including peer-reviewed academic publications, non-academic publications, conference presentations, stakeholder websites and social media. All participants will be notified of the outcome of the study by newsletter, press release and infographic.

Once a final prototype has been developed, a multi-centre feasibility study will be carried out to explore the acceptability and practicality of the DA for patients, carers and HCPs in practice, and to assess whether the DA shows promise of being beneficial for the intended population. The protocol for the feasibility study will be presented in a subsequent publication. On completion of the feasibility study, it is anticipated that there will be a DA that can be used in clinical practice with the aim of improving the decision-making process and quality of decision for individuals with MND deciding whether to have a gastrostomy. It is also hoped that national and regional MND guidelines will make reference to the tool. The project therefore has the potential for multifaceted impact by advancing patient care, improving service delivery and providing useful insights for the development of decision aids in the future.

Twitter @DiAMoNDstudy_

Contributors All of the named authors meet the ICMJE criteria for authorship and have contributed to the paper as follows:

Conception of the work: SW

Design of the work, including the study protocol: SW, RM, AH, SB, CF, AR-S, CM, CE, LR, DL, SW, AK, PW, MH, KM

Drafting of the protocol manuscript: RM, SW

Critical revision of the protocol manuscript: SW, RM, AH, SB, CF, AR-S, CM, CE, LR, DL, SW, AK, PW, MH, KM

Final approval of the version to be published: SW, RM, AH, SB, CF, AR-S, CM, CE, LR, DL, SW, AK, PW, MH, KM

Acknowledgements CJ McDermott is supported by the Sheffield NIHR BRC.

For peer review only

Funding This work was supported by Marie Curie and the Motor Neurone Disease Association grant numbers: MCRGS-20171219-8005 and 963-794 respectively.

Competing interests None declared.

Data statement Data available on request from the corresponding author.

REFERENCES

1 Hoppitt T, Pall H, Calvert M, et al. A systematic review of the incidence and prevalence of long-term neurological conditions in the UK. *Neuroepidemiology* 2011;36(1):19-28.
2 Hardiman O, van den Berg LH, Kiernan MC. Clinical diagnosis and management of amyotrophic lateral sclerosis. *Nat Rev Neurol* 2011;7(11):639-49.
3 Motor Neurone Disease Association. What is MND? 2018 [Available from: https://www.mndassociation.org/about-mnd/where-do-i-start/what-is-mnd/ Access date: June 2019].
4 Baumer D, Talbot K, Turner MR. Advances in motor neurone disease. *J R Soc Med* 2014;107(1):14-21.
5 Motor Neurone Disease Association. Dysphagia [Available from: https://www.mndassociation.org/forprofessionals/mndmanagement/dysphagia/ Access date: June 2019]
6 O'Rourke F, Vickers K, Upton C, et al. Swallowing and oropharyngeal dysphagia. *Clin Med* 2014;14(2):196-9.
7 Andersen PM, Borasio GD, Dengler R, et al. EFNS task force on management of amyotrophic lateral sclerosis: guidelines for diagnosing and clinical care of patients and relatives. *Eur J Neurol* 2005;12(12):921-38.
8 Miller RG, Jackson CE, Kasarskis EJ, et al. Practice parameter update: the care of the patient with amyotrophic lateral sclerosis: drug, nutritional, and respiratory therapies (an evidence-based review): report of the Quality Standards Subcommittee of the American Academy of Neurology. *Neurology* 2009;73(15):1218-26.
9 Katzberg HD, Benatar M. Enteral tube feeding for amyotrophic lateral sclerosis/motor neuron disease. *Cochrane Database of Systematic Reviews* 2011(1):CD004030.
10 Stavroulakis T, Baird WO, Baxter SK, et al. The impact of gastrostomy in motor neurone disease: challenges and benefits from a patient and carer perspective. *BMJ support* 2016;6(1):52-9.
11 ProGas Study Group. Gastrostomy in patients with amyotrophic lateral sclerosis (ProGas): a prospective cohort study. *Lancet Neurol* 2015;14(7):702-9.
12 National Institute for Health and Care Excellence (NICE). Motor neurone disease: assessment and management 2016 [Available from: https://www.nice.org.uk/guidance/ng42/chapter/recommendations#nutrition-and-gastrostomy Access date: June 2019]
13 Motor Neurone Disease Association. Living with MND: Swallowing, eating and drinking. 2019 [Available from: https://www.mndassociation.org/about-mnd/living-with-mnd/eating-and-drinking/ Access date: June 2019].
14 International Patient Decision Aid Standards (IPDAS) Collaboration. What are patient decision aids 2017 [Available from: http://ipdas.ohri.ca/what.html Access date: June 2019].
15 Hogden A, Greenfield D, Caga J, et al. Development of patient decision support tools for motor neuron disease using stakeholder consultation: a study protocol. *BMJ Open* 2016;6(4).
16 Alden DL, Friend J, Schapira M, et al. Cultural targeting and tailoring of shared decision making technology: a theoretical framework for improving the effectiveness of patient decision aids in culturally diverse groups. *Soc Sci Med* 2014;105:1-8.

For peer review only

17 Greenaway LP, Martin NH, Lawrence V. Accepting or declining non-invasive ventilation or gastrostomy in amyotrophic lateral sclerosis: patients' perspectives. *Journal of Neurology* 2015;262:1002-13.
18 Morris MA, Dudgeon BJ, Yorkston K. A qualitative study of adult AAC users' experiences communicating with medical providers. *Disabil Rehabil Assist Technol* 2013;8:472-81.
19 NHS England. Shared Decision Making [Available from: https://www.england.nhs.uk/shared-decision-making/ Access date: June 2019]
20 NHS England. Universal personalised care [Available from: https://www.england.nhs.uk/personalisedcare/upc/comprehensive-model/ Access date: June 2019]
21 NHS. The NHS Long Term Plan. 2019 [Available from: www.longtermplan.nhs.uk Access date: June 2019]
22 NHS Confederation. Taking the value-based agenda forward: The five essential components of value-based approaches to health and care. 2018 [Available from: https://www.nhsconfed.org/resources/2018/09/taking-the-value-based-agenda-forward Access date: June 2019]
23 Patton MQ. Qualitative Research and Evaluation Methods. Thousand Oaks, CA: Sage Publications; 2002.
24 Elwyn G, Kreuwel I, Durand MA, et al. How to develop web-based decision support interventions for patients: a process map. *Patient Educ Couns* 2011;82(2):260-5.
25 Elwyn G, et al on behalf of the International Patient Decision Aids Standards (IPDAS) Collaboration. Developing a quality criteria framework for patient decision aids: online international Delphi consensus process. *BMJ* 2006;333:417.
26 Medical Research Council. Developing and evaluating complex interventions: new guidance. *BMJ* 2008;337.
27 Centre for Reviews and Dissemination. Systematic reviews: CRD's guidance for undertaking reviews in health care 2009 [Available from: https://www.york.ac.uk/media/crd/Systematic_Reviews.pdf Access date: June 2019]
28 Reyna V. A Theory of Medical Decision Making and Health: Fuzzy Trace Theory. Med Decis Making 2008;28(6):850-865.
29 Paynter C, Cruice M, Mathers S, et al. Communication and cognitive impairments and health care decision making in MND: A narrative review. J Eval Clin Pract 2019 Jul Epub ahead of print
30 Hobson E, Fazal S, Shaw P, et al. “Anything that makes life’s journey better.” Exploring use of digital technology by people living with motor neurone disease. Amyotroph Lateral Scler Frontotemporal Degener 2017;18(5-6):378-387.
31 Gale NK, Heath G, Cameron E, et al. Using the framework method for the analysis of qualitative data in multidisciplinary health research. *BMC Med Res Methodol* 2013;13:117.
32 DSDM Agile Project Framework Handbook. MoSCoW Prioritisation. [Available from: https://www.agilebusiness.org/content/moscow-prioritisation Access date: June 2019]
33 Yardley L, Morrison LG, Andreou P, et al. Understanding reactions to an internet-delivered health-care intervention: accommodating user preferences for information provision. *BMC Med Inform Decis Mak* 2010;10(1):52.
34 NHS Health Research Authority. Consent and Participant Information Guidance: Principles of consent: General principles and role of Participant Information Sheets [Available from: http://www.hra-decisiontools.org.uk/consent/principles-general.html Access date: June 2019]

For peer review only

Appendix 1: Phase 1 Interview Topic Guides

1.1 Patient

Interview objective: to explore relevant personal experiences of people with MND deciding whether to have a gastrostomy, as well as getting their feedback on information that would be important to include in a decision aid, what a decision aid might look like and when it should be introduced.

Before starting: complete consent form.

Start the interview by providing a background to the study and purpose of the interview (based on PIS). Emphasise the research role and that the researcher will not be able to provide any clinical guidance or advice. Identify which term for gastrostomy patient is familiar with e.g. gastrostomy, PEG, RIG, feeding tube - to be used throughout the interview. Remind participants what a decision aid is.

- Start recording -

Please could you confirm whether or not you have a feeding tube at present?

If Yes use set 1 questions, if No use set 2 questions.

Questions – Set 1

1. After you were diagnosed, when were you introduced to the role of tube feeding in MND? Was this the right time for you?

2. Can you tell me about your experience of making the decision to have a feeding tube?
   a. When did you have the procedure?
   b. How did you feel during the decision making process?
   c. What made you go ahead with it?
   d. In hindsight, do you think you went ahead at the right time?

3. How did you go about making the decision to have a feeding tube?
   a. Who did you talk to? Prompts: HCP/family/friend/others with MND
   b. What information were you provided with? Prompts: verbal from your HCP, written information, websites
   c. What kind of information did you want? Prompts: procedure, effect on daily life, effect on progression of MND
   d. How much information did you want?
   e. Did you look for more information anywhere else? Prompts: Internet
      i. What did you think about the information you found?
      ii. Do you have any examples of websites you liked / disliked?
   f. Did you feel confident that you had all the information that you needed to make the decision, and did you understand it all?
   g. Is there anything you know now that you would have liked to have known before you made the decision to have a feeding tube?

For peer review only - http://bmjopen.bmj.com/site/about/guidelines.xhtml

Now I want to ask you a few questions on what information you think a decision aid might include, and what it might look like

4. Can you tell me what information you think people need to know before deciding whether to have a feeding tube?

5. Would you have been interested in using a decision aid to help choose whether to have a feeding tube? Why or why not?
   a. If yes, when would you have wanted to start using the decision aid?
   b. Would you like to use the decision aid by yourself or with someone else? Who? Why?
   c. Would you like to access the decision aid at home?
   d. What do you think the most important information is to include?
   e. How detailed do you think the information provided should be?
   f. How should the information be presented? Prompts: sound bites, video clips, images
   g. What type of device/s should this be available on? Computer, mobile
   h. How do you think you would use a decision aid in terms of the amount of time you would spend on it? Do you think you would use it more than once?

Finally, is there anything we haven't covered that you would like to add?

Thank you for taking part in the interview today.

Signposting if participants have concerns:

MNDA website: www.mndassociation.org

Suggest participant contacts MND care team or GP if they have any clinical questions.

Questions – Set 2

1. After you were diagnosed, when were you introduced to the role of tube feeding in MND? Was this the right time for you?

2. Have you decided whether or not to have a feeding tube? You may still be in the process of making this decision.

3. Can you tell me about your experience (+/- so far) of making this decision?
   a. How did (do) you feel during the decision making process?
   b. **If the participant has decided against a feeding tube** - What made you decide against it?
   c. **If the participant has decided against a feeding tube** - Do you think you will revisit this decision in the future?

4. [How did go / how are you going / how will you go] about deciding whether or not to have a feeding tube?
   a. Who [did/are/will] you talk/ing to? Prompts: HCP/family/friend/others with MND

For peer review only - http://bmjopen.bmj.com/site/about/guidelines.xhtml
For peer review only

b. What information [were you / have you been] provided with? Prompts: verbal from your HCP, written information, websites
c. What kind of information [did / do] you want? Prompts: procedure, effect on daily life, effect on progression of MND
d. How much information [did / do] you want?
e. [Did / will] you look for more information anywhere else? Prompts: Internet
   If the patient has already looked elsewhere for information:
   iii. What did you think about the information you found?
   iv. Do you have any examples of websites you liked / disliked?
f. [Did / do] you feel confident that you [had / have] all the information that you [needed / need] to make the decision, and [did / do] you understand it all?

Now I want to ask you a few questions on what information you think a decision aid might include, and what it might look like

5. Can you tell me what information you think people need to know before deciding whether to have a feeding tube?

6. Would you have been interested in using a decision aid to help choose whether to have a feeding tube? Why or why not?
   a. If yes, when would you have wanted to start using the decision aid?
   b. Would you like to use the decision aid by yourself or with someone else? Who? Why?
   c. Would you like to access the decision aid at home?
   d. What do you think the most important information is to include?
   e. How detailed do you think the information provided should be?
   f. How should the information be presented? Prompts: sound bites, video clips, images
   g. What type of device/s should this be available on? Computer, mobile
   h. How do you think you would use a decision aid in terms of the amount of time you would spend on it? Do you think you would use it more than once?

Finally, is there anything we haven't covered that you would like to add?

Thank you for taking part in the interview today.

Signposting if participants have concerns:

MNDA website: www.mndassociation.org

Suggest participant contacts MND care team or GP if they have any clinical questions.

For peer review only - http://bmjopen.bmj.com/site/about/guidelines.xhtml

1.2 Carer

Interview objective: to explore relevant personal experiences of people caring for individuals with MND deciding whether to have a gastrostomy, as well as getting their feedback on information that would be important to include in a decision aid, what a decision aid might look like and when it should be introduced.

Before starting: complete consent form, collect demographic data.

Start the interview by providing a background to the study and purpose of the interview (based on PIS). Emphasise the research role and that the researcher will not be able to provide any clinical guidance or advice. Identify which term for gastrostomy participant is familiar with e.g. gastrostomy, PEG, RIG, feeding tube - to be used throughout the interview. Remind participants what a decision aid is. Explain that the term carer will be used to describe their relationship with the person with MND, that we appreciate that this may not be the ideal term in all cases but it is used as a broad term for consistency.

- Start recording –

Are you happy for us to use the first name of the individual with MND that you care for, throughout the interview?

If Yes - Can I ask their first name? If No – Ok, we will refer to them as 'the individual that you care for'.

Please could you confirm whether or not [insert name] has a feeding tube at present?

If Yes use set 1 questions, if No use set 2 questions.

Questions – Set 1

1. After [insert name] was diagnosed with MND, when were you both introduced to the role of tube feeding in MND? Was this the right time for you both?

2. Can you tell me about your experience of supporting [insert name] to make the decision to have a feeding tube?

  a. When did [he/she] have the procedure?
  b. How did you feel during the decision making process?
  c. What made [insert name] go ahead with it?
  d. At the time, did you think [insert name] was making the right decision?
  e. In hindsight, do you think [he/she] went ahead at the right time?

3. How did you go about supporting [insert name] to make the decision to have a feeding tube?

  a. Who did you talk to? Prompts: HCP/family/friend/others with MND
  b. What information were you provided with? Prompts: verbal from your HCP, written information, websites
  c. What kind of information did you want for yourself? Prompts: procedure, effect on daily life, effect on progression of MND
  d. How much information did you want?
  e. Did you look for more information anywhere else? Prompts: Internet

For peer review only - http://bmjopen.bmj.com/site/about/guidelines.xhtml

v. What did you think about the information you found?
vi. Do you have any examples of websites you liked / disliked?

f. Did you feel confident that you had all the information that you needed support [insert name] to make the decision and that you knew enough about what was going to happen? Did you understand it all?
g. Is there anything you know now that you would have liked to have known before [insert name] had feeding tube placed?

Now I want to ask you a few questions on what information you think a decision aid might include, and what it might look like:

4. Can you tell me what information you think people with MND, along with their carers, need to know before deciding whether to have a feeding tube?

5. Would you have been interested in being present while [insert name] used a decision aid? Why or why not? If yes, would you like someone else also to be present? Who? Why?

6. Would you (also) have been interested in looking at a decision aid without [insert name]?

7. If yes, when would you have wanted to start using the decision aid?

8. Do you think it would be a good idea to include separate information, specifically aimed at carers, in the decision aid?

9. What do you think the most important information is to include?

10. How detailed do you think the information provided should be?

11. How should the information be presented? Prompts: sound bites, video clips, images

12. What type of device/s should this be available on? Computer, mobile

13. Would you like to access the decision aid at home?

14. How do you think you would use a decision aid in terms of the amount of time you would spend on it? Do you think you would use it more than once?

Finally, is there anything we haven't covered that you would like to add?

Thank you for taking part in the interview today.

Signposting if participants have concerns:

MNDA website: www.mndassociation.org

Suggest participant contacts MND care team or GP if they have any clinical questions.

For peer review only - http://bmjopen.bmj.com/site/about/guidelines.xhtml

Questions – Set 2

1. After [insert name] was diagnosed, when were you both introduced to the role of tube feeding in MND? Was this the right time for you both?

2. Has [insert name] decided whether or not to have a feeding tube? [He/she] may still be in the process of making this decision. How do you feel about this decision?

3. Can you tell me about your experience (+/- so far) in supporting [insert name] to make this decision? [He/she] may still be in the process of making this decision.

   a. How did (do) you feel during the decision making process?
   b. **If the individual the participant cares for has decided against a feeding tube** - What made [insert name] decide against it?
   c. **If the individual the participant cares for has decided against a feeding tube**
      - At the time, did you think [insert name] was making the right decision?
      - Do you think [he/she] will revisit this decision in the future?

4. [How did go / how are you going / how will you go] about supporting [insert name] to decide whether to have a feeding tube?

   a. Who [did/are/will] you talk/ing to? Prompts: HCP/family/friend/others with MND
   b. What information [were you / have you been] provided with? Prompts: verbal from your HCP, written information, websites
   c. What kind of information [did / do] you want for yourself? Prompts: procedure, effect on daily life, effect on progression of MND
   d. How much information [did / do] you want?
   e. [Did / will] you look for more information anywhere else? Prompts: Internet
      If the participant has already looked elsewhere for information:
         vii. What did you think about the information you found?
         viii. Do you have any examples of websites you liked / disliked?
   f. [Did / do] you feel confident that you [had / have] all the information that you [needed / need] to make the decision, and [did / do] you understand it all?

Now I want to ask you a few questions on what information you think a decision aid might include, and what it might look like:

5. Can you tell me what information you think people with MND and their carers need to know before deciding whether to have a feeding tube?

6. Would you have been interested in being present while [insert name] used a decision aid? Why or why not?

   a. If yes, would you like someone else also to be present? Who? Why?
   b. Would you (also) have been interested in looking at a decision aid without [insert name]?
   c. If yes, when would you have wanted to start using the decision aid?
   d. Do you think it would be a good idea to include separate information, specifically aimed at carers, in the decision aid?
   e. What do you think the most important information is to include?
   f. How detailed do you think the information provided should be?
   g. How should the information be presented? Prompts: sound bites, video clips, images
   h. What type of device/s should this be available on? Computer, mobile

For peer review only - http://bmjopen.bmj.com/site/about/guidelines.xhtml

i. Would you like to access the decision aid at home?
j. How do you think you would use a decision aid in terms of the amount of time you would spend on it? Do you think you would use it more than once?

Finally, is there anything we haven't covered that you would like to add?

Thank you for taking part in the interview today.

Signposting if participants have concerns:

MNDA website: www.mndassociation.org

Suggest participant contacts MND care team or GP if they have any clinical questions.

1.3 Healthcare Professional

Interview objective: to gain feedback from healthcare professionals working with individuals with MND, on what information is important to include in a decision aid for individuals with MND deciding whether to have a gastrostomy, what a decision aid might look like and when it should be introduced.

Before starting: complete consent form, collect demographic data.

Start the interview by providing a background to the study and purpose of the interview (based on PIS). Identify which term for gastrostomy participant is familiar with e.g. gastrostomy, PEG, RIG, feeding tube - to be used throughout the interview. Remind participants what a decision aid is.

- Start recording –

Questions

1. Can you tell me what information you think people with MND need to know before deciding whether to have a feeding tube?

2. Can you tell me what information you think carers of people with MND should know when supporting the individual they care for in this decision-making process, as well as for their own awareness and well-being?

3. Have you had experience of people with MND and their carers having different opinions about the use of feeding tubes? If yes, how was the situation resolved?

4. Do you believe people with MND (and their carers) would be interested in using a decision aid to support them to decide whether to have a feeding tube? Why or why not?

a. If yes, when should the decision aid be introduced?
b. Should the decision aid be used independently or with someone else? Who? Why?
c. Do you think it would be beneficial for people to be able to access the decision aid at home? Would you have any concerns about this?
d. What do you think is the most important information to include?
e. How detailed do you think the information provided should be?

For peer review only - http://bmjopen.bmj.com/site/about/guidelines.xhtml

f. How should the information be presented? Prompts: sound bites, video clips, images
g. What type of device/s should this be available on? Computer, mobile
h. How do you think you would use a decision aid in practice? Prompts: with staff supervision - yourself or another member of staff, amount of time, would you use it more than once with the same patient?

Finally, is there anything we haven't covered that you would like to add?

Thank you for taking part in the interview today.

For peer review only

For peer review only - http://bmjopen.bmj.com/site/about/guidelines.xhtml

Appendix 2: Phase 2 Think-Aloud Interview Guides

2.1 Patient

Think-Aloud interview objectives:

1. To examine how participants use and navigate the Decision Aid prototype and explore whether the information is presented in a way that the participants find easy and logical to access.
2. To examine whether the information is presented in a way in that participants understand.
3. To explore which aspects of the Decision Aid prototype participants find most/least useful.
4. To explore participants views about the design of the Decision Aid prototype, including colours, logos

Before interview: complete consent form

Start the interview by providing a background to the study and purpose of the interview (based on PIS). Emphasise the research role and that the researcher will not be able to provide any clinical guidance or advice. Remind participants what a decision aid is.

Provide explanation of what will happen next:

Once we begin recording I would like you to login in and use the patient section of the decision aid as you would normally, as if I was not here, and say your thoughts aloud. This can be about anything that you think of. To help you think aloud you may find it useful to read aloud or tell me what you are clicking on and why. You may find at times I will say aloud what you have clicked on or what page you are looking at – this is just so when I listen to your views again, I know what part of the website you are talking about. I may also ask you some simple questions to prompt your thoughts as you go.

This is not a test and you are not being judged. There are no right or wrong answers, so please say any thoughts that spring to mind, even if you think they might not be important. Please do feel free to say any negative thoughts you may have about the decision aid as these will be really useful in helping us improve it. I just want you to say out loud any thoughts which are running through your mind. After you have finished working through the decision aid, I will ask you some questions about the tool.

- Invite participant to ask questions
- Permission to start recording

- Start recording -

For peer review only - http://bmjopen.bmj.com/site/about/guidelines.xhtml

Part 1: Think-Aloud interview

- Allow participant to explore the patient section of the decision aid as they would normally and without interfering with this.
- Do not explain anything about the decision aid other than how to login.
- Prompt the participant to continue verbalising their thoughts as necessary. Allow participants to read information and gather their thoughts before prompting. If it appears that they are moving onto the next section, this may be an appropriate time to prompt.
- **Do not ask specific questions at this time.**

To start, please use the instruction sheet to login to the decision aid. Let me know about any thoughts you have as you do this. After logging in, please use the decision aid as you would normally.

Think-Aloud prompts:

- What are your first thoughts?
- What are your first thoughts about this page?
- What are you thinking now?
- What was going through your mind as you looked at that page?
- Can you tell me what you think about this page?
- What do you feel about [this activity; this information]?
- That's interesting. Can you tell me more about that?

Part 2: Retrospective evaluation questions

- What were your first thoughts about the Decision Aid?
- What did you think about the look of the Decision Aid?
- How easy was it to find the information you wanted? How easy was it to find your way around the site?
- What did you like about the decision aid?
- What did you dislike about the decision aid?
- What did you find most useful?
- What did you find least useful?
- How do you think the decision aid can be improved?
- Did you find any information confusing?

Finally, is there anything we haven't covered that you would like to add?

Thank you for taking part in the interview today.

Signposting if participants have concerns:

MNDA website: www.mndassociation.org

Suggest participant contacts MND care team or GP if they have any clinical questions.

For peer review only - http://bmjopen.bmj.com/site/about/guidelines.xhtml

For peer review only

2.2 Carer

Think-Aloud interview objectives:

5. To examine how participants use and navigate the Decision Aid prototype and explore whether the information is presented in a way that the participants find easy and logical to access.
6. To examine whether the information is presented in a way in that participants understand.
7. To explore which aspects of the Decision Aid prototype participants find most/least useful.
8. To explore participants views about the design of the Decision Aid prototype, including colours, logos

Before interview: complete consent form and collect demographic data

Start the interview by providing a background to the study and purpose of the interview (based on PIS). Emphasise the research role and that the researcher will not be able to provide any clinical guidance or advice. Remind participants what a decision aid is.

Provide explanation of what will happen next:

Once we begin recording I would like you to login and use the carers section of the decision aid as you would normally, as if I was not here, and say your thoughts aloud. This can be about anything that you think of. To help you think aloud you may find it useful to read aloud or tell me what you are clicking on and why. You may find at times I will say aloud what you have clicked on or what page you are looking at – this is just so when I listen to your views again, I know what part of the website you are talking about. I may also ask you some simple questions to prompt your thoughts as you go.

This is not a test and you are not being judged. There are no right or wrong answers, so please say any thoughts that spring to mind, even if you think they might not be important. Please do feel free to say any negative thoughts you may have about the decision aid as these will be really useful in helping us improve it. I just want you to say out loud any thoughts which are running through your mind. After you have finished working through the decision aid, I will ask you some questions about the tool.

- Invite participant to ask questions
- Permission to start recording

- Start recording -

For peer review only - http://bmjopen.bmj.com/site/about/guidelines.xhtml

Part 1: Think-Aloud interview

- Allow participant to explore the carers section of the decision aid as they would normally and without interfering with this.
- Do not explain anything other than how to login.
- Prompt the participant to continue verbalising their thoughts as necessary. Allow participants to read information and gather their thoughts before prompting. If it appears that they are moving onto the next section, this may be an appropriate time to prompt.
- **Do not ask specific questions at this time.**

To start, please use the instruction sheet to login to the decision aid. Let me know about any thoughts you have as you do this. Once you have logged in, please use the decision aid as you would normally.

Think-Aloud prompts:

- What are your first thoughts?
- What are your first thoughts about this page?
- What are you thinking now?
- What was going through your mind as you looked at that page?
- Can you tell me what you think about this page?
- What do you feel about [this activity; this information]?
- That's interesting. Can you tell me more about that?

Part 2: Retrospective evaluation questions

- What were your first thoughts about the Decision Aid?
- What did you think about the look of the Decision Aid?
- How easy was it to find the information you wanted? How easy was it to find your way around the site?
- What did you like about the decision aid?
- What did you dislike about the decision aid?
- What did you find most useful?
- What did you find least useful?
- How do you think the decision aid can be improved?
- Did you find any information confusing?

Finally, is there anything we haven't covered that you would like to add?

Thank you for taking part in the interview today.

Signposting if participants have concerns:

MNDA website: www.mndassociation.org

Suggest participant contacts MND care team or GP if they have any clinical questions.

For peer review only - http://bmjopen.bmj.com/site/about/guidelines.xhtml

2.3 Healthcare Professional

Phase 2 Think-Aloud Interview Guide – healthcare professional

Think-Aloud interview objectives:

9. To examine participants' experience of the usability and navigation of the Decision Aid prototype and explore whether they believe the information is presented in a way that they as well as patients and carers will find easy and logical to access.
10. To examine whether the information is presented in a way in that participants feel patients and carers will understand, whilst being clinically sound.
11. To explore which aspects of the Decision Aid prototype participants believe are most/least useful.
12. To explore participants views about the design of the Decision Aid prototype, including colours, logos

Before interview: complete consent form and collect demographic data

Start the interview by providing a background to the study and purpose of the interview (based on PIS). Remind participants what a decision aid is.

Provide explanation of what will happen next:

Once we begin recording I would like you to login and use the decision aid as you might do with a patient or carer, as if I was not here, and say your thoughts aloud. This can be about anything that you think of. To help you think aloud you may find it useful to read aloud or tell me what you are clicking on and why. You may find at times I will say aloud what you have clicked on or what page you are looking at – this is just so when I listen to your views again, I know what part of the website you are talking about. I may also ask you some simple questions to prompt your thoughts as you go.

There are no right or wrong answers, so please say any thoughts that spring to mind, even if you think they might not be important. Please do feel free to say any negative thoughts you may have about the decision aid as these will be really useful in helping us improve it. I just want you to say out loud any thoughts which are running through your mind. After you have finished working through the decision aid, I will ask you some questions about the tool.

- Invite participant to ask questions
- Permission to start recording

- Start recording –

For peer review only - http://bmjopen.bmj.com/site/about/guidelines.xhtml

Part 1: Think-Aloud interview

- Allow participant to explore the decision aid as they would normally and without interfering with this.
- Do not explain anything other than how to login.
- Prompt the participant to continue verbalising their thoughts as necessary. Allow participants to read information and gather their thoughts before prompting. If it appears that they are moving onto the next section, this may be an appropriate time to prompt.
- **Do not ask specific questions at this time.**

To start, please use the instruction sheet to login to the decision aid. Let me know about any thoughts you have as you do this. Once you have logged in, please use the decision aid as you would normally.

For peer review only

Think-Aloud prompts:

- What are your first thoughts?
- What are your first thoughts about this page?
- What are you thinking now?
- What was going through your mind as you looked at that page?
- Can you tell me what you think about this page?
- What do you feel about [this activity; this information]?
- That's interesting. Can you tell me more about that?

Part 2: Retrospective evaluation questions

- What were your first thoughts about the Decision Aid?
- What did you think about the look of the Decision Aid?
- How easy was it to find the information you wanted? How easy was it to find your way around the site?
- What did you like about the decision aid?
- What did you dislike about the decision aid?
- What did you find most useful?
- What did you find least useful?
- How do you think the decision aid can be improved?
- Did you find any information confusing?

Finally, is there anything we haven't covered that you would like to add?

Thank you for taking part in the interview today.

For peer review only - http://bmjopen.bmj.com/site/about/guidelines.xhtml

DEVELOPING A WEB-BASED PATIENT DECISION AID FOR GASTROSTOMY IN MOTOR NEURONE DISEASE: A STUDY PROTOCOL

Journal:	*BMJ Open*
Manuscript ID	bmjopen-2019-032364.R2
Article Type:	Protocol
Date Submitted by the Author:	29-Oct-2019
Complete List of Authors:	Maunsell, Rose; University of Southampton, Health Sciences<br>Bloomfield, Suzanne; Countess Mountbatten Hospice, Southampton<br>Erridge, Clare; Southampton General Hospital<br>Foster, Claire; University of Southampton, Health Sciences<br>Hardcastle, Maggi; Rowans Hospice<br>Hogden, Anne; University of Tasmania, Australian Institute of Health Service Management<br>Kidd, Alison; University of Southampton, Carer representative<br>Lisiecka, Dominika; University of Limerick, School of Allied Health<br>McDermott, Christopher; university of sheffield,<br>Morrison, Karen; University of Southampton, Faculty of Medicine<br>Recio-Saucedo, Alejandra; University of Southampton<br>Rickenbach, Louise; Motor Neurone Disease Association<br>White, Sean; Sheffield Teaching Hospitals NHS Foundation Trust, Dietetic Department<br>Williams, Peter; University of Southampton, Patient representative<br>Wheelwright, Sally J.; Univ Southampton, Health Sciences
**Primary Subject Heading**:	Neurology
Secondary Subject Heading:	Nutrition and metabolism, Qualitative research, Research methods
Keywords:	Motor neurone disease < NEUROLOGY, NUTRITION & DIETETICS, Adult palliative care < PALLIATIVE CARE, Protocols & guidelines < HEALTH SERVICES ADMINISTRATION & MANAGEMENT

SCHOLARONE™ Manuscripts

DEVELOPING A WEB-BASED PATIENT DECISION AID FOR GASTROSTOMY IN MOTOR NEURONE DISEASE: A STUDY PROTOCOL

Corresponding Author

Dr Sally Wheelwright
Health Sciences,
University Of Southampton,
SO17 1BJ
Email: S.J.Wheelwright@soton.ac.uk
Tel: 02380 597860

Co-authors

Mrs Rose Maunsell
Health Sciences,
University Of Southampton,
SO17 1BJ
Email: r.evill@soton.ac.uk
Tel: 02380 597939

Ms Suzanne Bloomfield
Community CNS in Palliative Care Team
Countess Mountbatten Hospice
Southampton, SO30 3JB
Tel: 02380 475874
Email: suzanne.bloomfield@uhs.nhs.uk

Ms Clare Erridge
Lead Motor Neurone Disease Care Centre Coordinator Mailpoint 101 Wessex Neurological Centre
Southampton General Hospital
Tremona Road
Southampton, SO16 6YD
Tel: 02381 206544
Email: clare.erridge@uhs.nhs.uk

Professor Claire Foster
Professor of Psychosocial Oncology
School of Health Sciences
University of Southampton
SO17 1 BJ
Tel: (023) 8059 4006
Email: C.L.Foster@soton.ac.uk

Ms Maggi Hardcastle
Clinical Specialist Physiotherapist
The Rowans Hospice
Purbrook Heath Road
Waterlooville, PO7 5RU
Tel: 02392 250001
Email: maggi.hardcastle@rowanshospice.co.uk

Dr Anne Hogden
Senior Lecturer

For peer review only

Australian Institute of Health Service Management
University of Tasmania, Rozelle Campus
Cnr Glover and Church Streets, Lilyfield, NSW 2040
Tel: +61 2 8572 7922
Email: anne.hogden@utas.edu.au

Dr Alison Kidd
Lay person
Corlan, Pencelli, Brecon, Powys, LD3 7LX
Tel: 091874 665401
Email: alison@prospectory.co.uk

Dr Dominika Lisiecka
Lecturer in Speech and Language Therapy
School of Allied Health
Health Sciences Building
Room H2-021
University of Limerick
Email: dominika.lisiecka@ul.ie

Professor Christopher McDermott
Professor of Translational Neurology / Consultant Neurologist
Sheffield Institute for Translational Neuroscience
385a Glossop Rd
Sheffield, S10 2HQ
Tel: 0114 2222295 (Secretary)
Email: c.j.mcdermott@sheffield.ac.uk

Professor Karen Morrison
Associate Dean for Education and Professor of Neurology
Faculty of Medicine, University of Southampton
Room AB217, Level B, South Academic Block, MP801, Southampton General Hospital, SO16 6YD
Tel: 02381 208134
Email: K.E.Morrison@soton.ac.uk

Dr Alejandra Recio-Saucedo
Research Fellow, Portfolio Insight and Publications
NIHR Evaluation, Trials and Studies Coordinating Centre (NETSCC)
University of Southampton
Alpha House
Enterprise Road
Southampton, SO16 7NS
Tel: 02380 595606
Email: ars@ecs.soton.ac.uk

Ms Louise Rickenbach
Regional Care Development Adviser for South Central
Motor Neurone Disease Association
Tel: 03453751831
Email: louise.rickenbach@mndassociation.org

Mr Sean White
Dietetic Department
Nurses Home
Northern General Hospital

For peer review only

Herries Road
Sheffield S5 7AU
Tel: 0114 2714162
E-mail: sean.white@sth.nhs.uk

Mr Peter Williams
Lay Person
Corlan, Pencelli, Brecon, Powys, LD3 7LX
Tel: 091874 665401
Email: peter@prospectory.co.uk

Dr Sally Wheelwright
Senior Research Fellow
School of Health Sciences
University of Southampton
SO17 1BJ
Tel: (023) 8059 7860
Email: S.J.Wheelwright@soton.ac.uk

For peer review only

Word count, excluding title page, abstract, references, figures and tables: 4207

DEVELOPING A WEB-BASED PATIENT DECISION AID FOR GASTROSTOMY IN MOTOR NEURONE DISEASE: A STUDY PROTOCOL

Rose Maunsell[1], Suzanne Bloomfield[2], Clare Erridge[3], Claire Foster[1], Maggi Hardcastle[4], Anne Hogden[5], Alison Kidd[6], Dominika Lisiecka[7], Christopher J McDermott[8], Karen Morrison[9], Alejandra Recio-Saucedo[10], Louise Rickenbach[11], Sean White[12], Peter Williams[13] and Sally Wheelwright[1]

[1]Health Sciences, University of Southampton, United Kingdom [2]Countess Mountbatten Hospice, Southampton, United Kingdom [3]Southampton General Hospital, Southampton, United Kingdom [4]The Rowans Hospice, Waterlooville, United Kingdom [5]Australian Institute of Health Service Management, University of Tasmania, Australia [6]Carer representative, University of Southampton, United Kingdom [7]School of Allied Health, University of Limerick, Ireland [8]University of Sheffield, United Kingdom [9]Faculty of Medicine, University of Southampton, United Kingdom [10]NIHR Evaluation, Trials and Studies Coordinating Centre (NETSCC), University of Southampton, United Kingdom [11]Motor Neurone Disease Association, Northampton, United Kingdom [12]Sheffield Teaching Hospitals NHS Foundation Trust, Sheffield, United Kingdom [13]Patient representative, University of Southampton, United Kingdom

ABSTRACT

Introduction:

Motor neurone disease (MND) is a progressive, incurable disease, characterised by degeneration of the nerves in the brain and spinal cord. Due to the multisystem effects of the disease, patients are faced with many complex, time sensitive decisions, one of which is the decision on gastrostomy feeding. There are currently no published decision aids to support patients making this decision in the UK. This study will develop and pilot a patient decision aid to: provide evidence-based information on gastrostomy placement and feeding that is relevant to people with MND; communicate the risks and benefits associated with each option; check understanding; and clarify personal values and preferences, enabling patients to make a decision congruent with their values and appropriate for them.

Methods and analysis:

A two phase process, observing the International Patient Decision Aid Standards (IPDAS), will be used to develop the decision aid, over 24 months starting January 2019. Phase 1 will use literature reviews and stakeholder interviews and surveys to identify essential content for the decision aid, and explore the best way to present this. In the second phase, a prototype decision aid will be developed and revised using stakeholder feedback in an iterative process. Stakeholders will include individuals with MND, their carers and the healthcare professionals (HCPs) working with them.

Ethics and dissemination:

Ethical approval for the study has been granted by West of Scotland Research Ethics Service, reference 19/WS/0078. Study findings will be disseminated through academic and non-academic publications, conference presentations, stakeholder websites and social media. A feasibility study will follow, to explore the acceptability and practicality of the decision aid for patients, carers and HCPs in practice and to assess whether the decision aid shows promise of being beneficial for the intended population.

Keywords: motor neuron disease, gastrostomy, enteral nutrition, decision making, decision support techniques

Strengths and limitations of this study

Review of the literature will ensure all relevant evidence is captured.

A user-centric approach to development will result in a decision aid that addresses the needs of patients with motor neurone disease (MND).

Using an iterative process to determine essential content for the decision aid and to develop the prototype may be time consuming.

Allowing patients the option to have a family member, friend or carer present during the interviews has the potential to influence the honesty and openness of their responses.

INTRODUCTION

Motor neurone disease (MND) is a progressive, incurable disease, characterised by degeneration of the nerves in the brain and spinal cord, leading to muscle weakness. It is estimated that up to 5,000 people are living with MND at any one time in the UK, with reported incidence rates of 1.06 to 2.4 per 100,000 people per year(1). Typical life expectancy from symptom onset is 3 years(2). However, due to the heterogeneity of MND, prognosis is unpredictable, with some individuals living for many years after diagnosis and others far less(3). Patients will experience volatile multi-system physical deterioration over the course of their disease, which may be accompanied by cognitive and behavioural change(4), requiring them to navigate a challenging care pathway with numerous complex decisions.

Dysphagia (difficulty swallowing) is common in people with MND. Those with bulbar subtypes of the disease will typically experience dysphagia early on in their disease trajectory, with more than two-thirds of all people with MND affected in late stages of the disease(5). Dysphagia may cause

For peer review only

distressing coughing and choking, laboured mealtimes, and aspiration, which in turn can lead to recurrent chest infections, nutritional decline and weight loss(6).

When an individual with MND presents with the signs and symptoms of dysphagia, gastrostomy tube feeding is often recommended, based on the assumption that there will be a beneficial impact on nutritional outcome, survival and quality of life (QOL)(7, 8). Gastrostomy refers to the opening made in the abdomen, through which the feeding tube enters the stomach, and as with any surgical procedure, carries a level of risk. However, there are no randomised controlled trials (RCTs) that explore the efficacy of tube feeding in MND and results from other research is equivocal, weak or lacking with respect to survival, nutritional outcome and QOL respectively(9).

Gastrostomy feeding can have a significant impact on an individual's everyday life, with both positive and negative consequences(10). Negative impacts include difficulties with feed administration, handling the equipment and managing supplies and storage, along with potential clinical complications and the psychological impact, such as anxiety around the tube and social isolation through loss of shared mealtimes. Potential benefits may include relief of anxiety associated with prolonged, effortful mealtimes by removing the need to eat and drink, weight stabilisation and perceived survival benefit. From the patient and carer perspective, this means the decision regarding whether to have a gastrostomy is not straightforward. To add to the complexity, there is also a time sensitivity to this decision, which was explored in ProGas(11), a large longitudinal prospective cohort study that investigated optimum timing and method of gastrostomy for people with MND. The study found that while earlier gastrostomy may improve survival time and nutritional outcomes, some patients might delay placement or not want a tube placing at all. This is reflected in current National Institute for Health and Care Excellence (NICE) guidelines for MND(12), which state that the role of gastrostomy feeding should be discussed at an early stage but that it must be recognised that not all individuals will wish to proceed.

Whilst there are some excellent educational resources about gastrostomy placement and feeding, including 'myTube', informed by the findings of ProGas(11) and online information provided by the MND Association(13), there are no published patient decision aids (DAs) for this group in the UK. DAs are designed to do more than provide information; they are usually developed for complex, preference based decisions that require greater consideration(14). These decisions will often have multiple options with features or outcomes that people value differently, in this case: undergoing gastrostomy placement, deciding against gastrostomy, or deciding to revisit the issue at a later date. Often the evidence-base for each option is limited and so the best choice depends on the personal importance the individual places on the benefits, harms, burden and scientific uncertainties(14). By presenting evidence-based information, communicating the risks and benefits associated with each option, checking understanding and clarifying personal values and preferences, a DA may help individuals with MND make a decision about whether a gastrostomy is congruent with their values and appropriate for them(15). They can also provide an outline for conversations with family members and healthcare professionals, and encourage a collaborative approach to decision-making(16). It is crucial to acknowledge the importance of involving close family members and carers in the gastrostomy decision-making process. Research has shown that carers may directly or indirectly influence patient decision-making and that patients often want to share the burden of decision-making with family and carers(17), in some cases relying on the carer or family member in order to participate in the decision-making process(18). For individuals with MND, communication difficulties secondary to their disease may augment their reliance on carers or family members. Whilst the values and preferences of the patient must take priority, developing a DA that includes content for carers ensures that these individuals are well informed and able to participate fully in

discussions, thus offering the patient better support in the decision-making process. It also provides support for the carer themselves; allowing them to prepare for the potential impact of the patient's decision on their own lives and providing sign posting to further support and information.

The development of a DA to support shared decision-making aligns with current NHS priorities of patient centred, value-based healthcare. Shared decision-making is a key component of NHS England's personalised care approach, laid out in their Universal personalised care model(19, 20), as part of the 10 Year Plan(21). The model is underpinned by evidence that shared decision-making leads to more realistic expectations, a better match between individuals' values and treatment choices, and fewer unnecessary interventions(20). NHS Rightcare's value-based initiative(22) also highlights the importance of listening to patients to identify what is of value to them to ensure that their care 'delivers' on an individual level, and using this to design and deliver services accordingly. The DA will support these NHS approaches by empowering the patient to participate in shared decision-making regarding gastrostomy placement.

The study will answer the following research questions:

1. What information should be included in a DA for people with MND considering whether to have a gastrostomy, their carers and health care professionals, and how is this best presented?

2. Do people with MND, carers and health care professionals find a web-based DA easy to use and useful?

METHODS AND ANALYSIS

This research protocol details the process for the development and piloting of a decision aid (DA) for individuals with motor neurone disease (MND), deciding whether to have a gastrostomy tube for feeding. The study will be conducted over 24 months and started in January 2019.

The study will have two phases:

Phase 1: will address the first research question by investigating what information should be included in the DA and how this is best presented.

Phase 2: the prototype DA will be developed, and the second research question will be addressed by exploring whether the prototype DA is easy to use and useful to the end user/s. Usefulness is defined in terms of whether it meets users' needs, attitude towards using it and perceived benefit.

This will be followed by a multi-centre feasibility study (Phase 3) to explore the acceptability and practicality of the DA for patients, carers and HCPs in practice, and to assess whether the DA shows promise of being beneficial for the intended population. Details of which will be presented in a later publication.

Participants

Participants will include individuals from the three key stakeholder groups involved in the decision-making process: individuals living with MND (patients), their carers and the healthcare professionals working with them.

Purposive sampling(23) will be employed to optimise stakeholder representation, ensuring the decision aid (DA) content meets the needs of the UK MND population. The sampling frame will be based on time since diagnosis and gastrostomy placement (Table 1). Those participants in the 'no gastrostomy' group will include patients who have declined gastrostomy. A similar number of

participants will be sought in each of the green cells. Although recruitment of patients and carers in the amber cell will be attempted, it is anticipated that the number of patients and carers that fall into this group will be small.

Table 1: Patient and carer sampling frame

	<6 months since diagnosis	>6 months since diagnosis
Gastrostomy	Aim to recruit where possible	Interview: patients n= 10, carers n= 5<br>Beta-testing: patients n= 3-4, carers n= 3-4
No gastrostomy	Interview: patients n= 10, carers n= 5<br>Beta-testing: patients n=3-4, carers n= 3-4	Interview: patients n= 10, carers n= 5<br>Beta-testing: patients n=3-4, carers n= 3-4

We aim to recruit patients who are broadly representative of the MND population in terms of age, gender and subtype of MND and we will include both people who currently live alone and those who live with others. Health care professionals will be drawn from a variety of professions including doctors, nurses and allied health professionals.

An inclusion and exclusion criteria will be applied to potential participants (Table 2). The clinical care team will be consulted to confirm whether the individual has capacity to provide informed consent. Individuals assessed as lacking capacity and their carers will be excluded because their decision-making informational needs are likely to differ significantly from those with capacity. Future research should investigate the needs of individuals with MND lacking capacity, and their care network, in gastrostomy decision making.

Table 2: Inclusion and exclusion criteria

**Inclusion criteria**	**Exclusion criteria**
Individuals with a confirmed diagnosis of motor neurone disease (MND) with a life expectancy of at least a year as reported by the clinical team<br><br>OR<br>Carers, defined as 'Any adult who looks after a family member, partner or friend with motor neurone disease. The care they give is unpaid, and they have face-to-face contact with the person with MND at least 3 times a week' (currently or within the last year). They may or may not be in receipt of carers allowance.<br><br>OR<br>Healthcare professionals working with individuals with MND, involved in the gastrostomy decision making process or supporting patients with gastrostomy feeding<br><br>AND<br>Able to communicate, either verbally or using other means e.g. using a communication aid, at phrase or sentence level	Unable to participate in English<br><br><18 years<br><br>Individuals assessed as lacking capacity by the clinical team<br><br>Carers of individuals assessed as lacking capacity

Design

The methods for the two phases of the study are based on a validated model for web-based decision aids(24) and the original 2005 International Patient Decision Aid Standards(25), and will observe the Medical Research Council's guidance for the development of complex interventions(26). Phase 1 of the study will elicit the information that should be included in the decision aid (DA) and identify the

For peer review only

best way to present it. In the Phase 2, the DA will be developed and revised. A study advisory committee (SAC) including individuals living with MND, their carers, and healthcare professionals and researchers with experience in MND and/or patient decision-making has been assembled and has contributed to the study design.

Phase 1: Content and Presentation

Literature reviews

A literature review to identify qualitative studies that explore the informational needs of people with neurodegenerative disease considering a gastrostomy will be conducted using standard methods(27). The search will be limited to decision-making around gastrostomy, rather than including all healthcare decisions for this group, primarily because of the invasive nature of the procedure. The search will also be limited to studies of patients with neurodegenerative disease, rather than any condition where tube feeding may be offered, because of the parallels with MND experience and disease outcomes. Thematic analysis will be used to identify information that is valued when decision-making.

A second literature review will identify theories which could be used to underpin the DA and also the best methods for presenting information within the context of a web-based DA. This review will search for web-based DAs and studies describing their development and evaluation, both for patients with MND and other patient groups. Methods for presenting information will be tabulated, along with any information about efficacy. Information about the theoretical underpinning of DAs will also be collated to help with the selection of a theory to underpin the DA and to identify the active ingredients of the decision-making process. Final theory selection will be driven by the findings from the interviews. For example, if people want to get the 'gist' in the first layer of information, then delve deeper for more detail at their discretion, the Fuzzy Trace Theory (28) may be applied. The intention is to publish both literature reviews in a separate publication(s).

Interviews

Cross-sectional, semi-structured in-depth interviews with patient, carer and healthcare professional participants, will explore relevant personal experiences (individuals and carers only); important information to include when making a decision about whether to have a gastrostomy; what a DA might look like and when and how a DA should be introduced. The informational needs of carers supporting individuals to make this decision will also be investigated. An interview guide, developed with support from the SAC, will be used to guide but not constrain the interviews. Interviews will be carried out either in person or by telephone, at a time and place convenient to the participant. Interviews will be audio recorded and transcribed verbatim. It is recognised that a significant proportion of patient participants will have communication difficulties(29). To minimise the burden of the interviews for patients, they will be sent the interview guide ahead of the interview, to allow them to begin to construct their answers. Patients may respond verbally, or by using an alternative form of communication. This may include electronic communication devices, such as a smartphone or tablet device(30), or in writing (e.g. in person or via email). Patient and carer interviews will be conducted independently, although patient participants will be able to have a family member, friend or carer present in the interview if preferred.

Clinical (patients only) and demographic data will also be collected, during or prior to the interview, to allow description of the study sample and evaluation of whether it is representative of a typical MND population in the UK. Patient data will be obtained from medical notes, while demographic data from carers and healthcare professionals will be reported by the participant. This will include:

i. Patients: age, gender, ethnicity, level of education, employment status, domestic status, family history of MND, date of diagnosis, type of MND, site of onset, stage of disease, gastrostomy placed (yes or no, date if applicable and why)

ii. Carers: age, gender, ethnicity, level of education, employment status, family history of MND

iii. Healthcare professionals: years qualified, job title, time working in the field of MND.

Sample sizes for the interviews were chosen based on previous similar projects that have achieved data saturation with these sample sizes(31). We will evaluate this assumption during the analysis phase by checking that the final three interviews did not generate any new themes, if this criterion is not met, we will continue to carry out interviews until it is(32).

Decision Aid Content

Interview and literature review outputs will be synthesised using a framework analysis approach(33). One researcher (RM) will code each transcript, line by line, with a second researcher (SW) independently coding 10% of the transcripts. The coding system will be harmonised through discussion and the agreed coding system will then be applied to the whole dataset, with refinement as needed. Themes will be identified by grouping together similar codes. The coding system, along with the themes identified from the transcripts and the first literature review will also be discussed with the SAC and modified if necessary, until consensus is reached. NVivo 12 will be used for data management.

The identified themes will be presented in a survey as pieces of information which could be included in the DA. The survey will be sent to the participants who were interviewed, as well as members of the SAC and study team, who will all be asked to prioritise each suggested piece of content using the prioritisation model MoSCoW(34). For each piece of content, respondents will be asked to rate how crucial it is for this to be included in the DA (M– Must have; S – Should have; C – Could have; W– Would like if time permits). Respondents will choose whether to complete the survey online, on a hard copy or over the telephone.

Finally, a systematic search of the evidence base relating to each suggested piece of content will be carried out to ensure all information included in the DA is accurate. The search will include published papers, guidelines, best practice documents, other grey literature and input from clinical experts on the SAC.

Phase 2: Prototype development and piloting of the decision aid

Results from Phase 1 will inform the development of the prototype DA. The SAC and research team will use the findings from the interviews to select the relevant theory from those identified in the literature review, to underpin the DA. The SAC and research team will develop a prototype DA, using an iterative process, by incorporating each piece of information receiving the highest prioritisation scores in the Phase 1 survey and observing the 2005 IPDAS checklist (25). The IPDAS checklist details required content to ensure information about options is presented in sufficient detail for decision-making, that information is presented in an unbiased and understandable way, and that methods for clarifying and expressing patients' values are included, along with tools to support making the actual decision and communicating this decision to others.

The DA will be web-based, programmed using responsive web design, enabling use on tablets, laptops and smart phones. The prototype will then undergo alpha testing; where phase 1 study participants will review the prototype and complete a survey to provide feedback on:

1. Clarity of prototype information
2. Presentation and functionality of prototype information (user friendliness and acceptability)
3. Perceptions of utility

It is recognised that between the interview and alpha testing (expected timeframe of 3-8 months depending on when the participant is recruited), a proportion of patient participants may become too unwell to participate, or will die. The period between interview and alpha testing will be kept as short as possible, however if a patient is unable to participate in the alpha testing this will be treated as missing data.

For peer review only

Feedback will be discussed with the SAC and a second version of the prototype DA will then be produced. Version 2 will undergo beta testing; newly recruited patients, carers and healthcare professionals (n=5 of each) will talk through their use of the DA following the "think aloud" method(35). A think aloud interview guide will be used to prompt participants to verbalise their thoughts as they use the DA, allowing the assessment of usability, satisfaction and acceptability(35). Immediately after this, evaluation questions will be used to ascertain users' attitudes towards using the DA, whether it meets their needs and whether they think it is of benefit.

The SAC and study team will then meet again to review the results and produce version 3 of the prototype, which will undergo beta testing with a new set of participants (n=5 of each group). Finally, the SAC and study team will decide whether any additional changes are required and the final prototype will be produced.

The formal evaluation of the DA will take place in Phase 3, the details of which will be presented in a subsequent publication. In brief, the DA will be used in clinical settings. An evaluation framework, comprising measures of both the quality of the decision-making process and the quality of the decision itself will be developed and used to compare the outcomes of patients who use the DA and those who do not. This will be supplemented with semi-structured interviews with stakeholders to explore acceptability and practicality of the DA, and to provide insight into implementation.

Recruitment

Patient and carer participants will be identified by a member of their clinical care team in NHS MND and palliative care clinics across the Wessex and Sheffield regions. Permission will be obtained to share the contact details of any individual interested in participating, with the research team. Support groups, such as those run by the Motor Neurone Disease Association, will provide another avenue for the recruitment of patients and carers. A member of the research team will attend the local support group in person to introduce the study, provide a participant information sheet to those interested in participating and request permission to make follow up contact. A third channel for patient and carer recruitment will be advertisements placed on relevant websites and social media platforms e.g. Twitter. Interested individuals will be invited to contact the researcher, who will provide a participant information sheet and request permission to make follow up contact. The clinical care team will be consulted to confirm the eligibility of all patient participants.

Eligible healthcare professionals will be identified either following direct approach by the researcher, via a member of the advisory committee (who will obtain consent to share their contact details with the researcher), or through adverts via social media and professional groups. A researcher will discuss the study with the individual, provide a participant information sheet and request permission to make follow up contact.

All participants will be asked to provide informed consent prior to participation in the study.

Patient and public involvement

People living with MND and their carers will be involved throughout the research process. The idea of developing a decision aid for people with MND considering a gastrostomy was initially presented to an MND support group for feedback and to invite members to join the research team.

Following award of the grant, people living with MND and their carers have joined the SAC; through this they will help to prioritise content for the DA, evaluate each iteration of the DA and comment on

For peer review only

all patient and carer facing study documents. They will also advise on the practicalities of including people with MND as research participants and have input on the recruitment strategy.

The views and insights of patient and carer participants will be central to the research and will form the majority of the study sample. Participants will be asked whether they wish to be kept up to date with the project's progress and those who do will be sent a newsletter.

ETHICS AND DISSEMINATION

Ethical approval for the study has been granted by West of Scotland Research Ethics Service, reference 19/WS/0078. Participant information sheets, consent forms, recruitment advertisements and GP letters were also approved. All amendments to study documents will be submitted in IRAS for HRA and REC approval.

Consent

The researcher will obtain written informed consent from participants prior to them entering into the study. For patient participants, this will include seeking permission to inform their GP of their participation in the study. In the case that the individual is unable to provide written consent, consent will be indicated by the participant answering 'yes' or 'no', either verbally or via a communication device, after each statement on the consent form. The form will then be signed by the parties involved in the consent conversation(36).

Given the sensitive nature of the topics to be discussed, the researcher will make clear that participation is voluntary, and the right to ask any questions and to decline participation at any time will be highlighted during the data collection.

Participant Confidentiality

The study will adhere to the principles of Good Clinical Practice (GCP) and in accordance with current Data Protection Regulations, the Data Protection Act 2018 (GDPR) and all other regulatory requirements, as appropriate. Necessary steps will be taken to ensure all data remain confidential at all times. Study data will be kept for 10 years from the end of the study, in accordance with University of Southampton policy. Only members of the research team will have access to the full dataset.

Dissemination

A dissemination and impact plan will be developed in the initial stages of the study. Dissemination will be led by working collaboratively with Marie Curie and the Motor Neurone Disease Association (MNDA) (the Funders) and Public Policy, University of Southampton, to develop and take forward the impact activities to ensure maximum publicity and benefit. Study findings will be disseminated via multiple channels, including peer-reviewed academic publications, non-academic publications, conference presentations, stakeholder websites and social media. All participants will be notified of the outcome of the study by newsletter, press release and infographic.

Once a final prototype has been developed, a multi-centre feasibility study will be carried out to explore the acceptability and practicality of the DA for patients, carers and HCPs in practice, and to assess whether the DA shows promise of being beneficial for the intended population. The protocol for the feasibility study will be presented in a subsequent publication. On completion of the feasibility study, it is anticipated that there will be a DA that can be used in clinical practice with the aim of improving the decision-making process and quality of decision for individuals with MND deciding whether to have a gastrostomy. It is also hoped that national and regional MND guidelines will make reference to the tool. The project therefore has the potential for multifaceted impact by

For peer review only

advancing patient care, improving service delivery and providing useful insights for the development of decision aids in the future.

Twitter @DiAMoNDstudy_

Contributors All of the named authors meet the ICMJE criteria for authorship and have contributed to the paper as follows:

Conception of the work: SW

Design of the work, including the study protocol: SW, RM, AH, SB, CF, AR-S, CM, CE, LR, DL, SW, AK, PW, MH, KM

Drafting of the protocol manuscript: RM, SW

Critical revision of the protocol manuscript: SW, RM, AH, SB, CF, AR-S, CM, CE, LR, DL, SW, AK, PW, MH, KM

Final approval of the version to be published: SW, RM, AH, SB, CF, AR-S, CM, CE, LR, DL, SW, AK, PW, MH, KM

Acknowledgements CJ McDermott is supported by the Sheffield NIHR BRC.

Funding This work was supported by Marie Curie and the Motor Neurone Disease Association grant numbers: MCRGS-20171219-8005 and 963-794 respectively.

Competing interests None declared.

Data statement Data available on request from the corresponding author.

For peer review only

REFERENCES

1 Hoppitt T, Pall H, Calvert M, et al. A systematic review of the incidence and prevalence of long-term neurological conditions in the UK. *Neuroepidemiology* 2011;36(1):19-28.
2 Hardiman O, van den Berg LH, Kiernan MC. Clinical diagnosis and management of amyotrophic lateral sclerosis. *Nat Rev Neurol* 2011;7(11):639-49.
3 Motor Neurone Disease Association. What is MND? 2018 [Available from: https://www.mndassociation.org/about-mnd/where-do-i-start/what-is-mnd/ Access date: June 2019].
4 Baumer D, Talbot K, Turner MR. Advances in motor neurone disease. *J R Soc Med* 2014;107(1):14-21.
5 Motor Neurone Disease Association. Dysphagia [Available from: https://www.mndassociation.org/forprofessionals/mndmanagement/dysphagia/ Access date: June 2019]
6 O'Rourke F, Vickers K, Upton C, et al. Swallowing and oropharyngeal dysphagia. *Clin Med* 2014;14(2):196-9.
7 Andersen PM, Borasio GD, Dengler R, et al. EFNS task force on management of amyotrophic lateral sclerosis: guidelines for diagnosing and clinical care of patients and relatives. *Eur J Neurol* 2005;12(12):921-38.
8 Miller RG, Jackson CE, Kasarskis EJ, et al. Practice parameter update: the care of the patient with amyotrophic lateral sclerosis: drug, nutritional, and respiratory therapies (an evidence-based

review): report of the Quality Standards Subcommittee of the American Academy of Neurology. *Neurology* 2009;73(15):1218-26.
9 Katzberg HD, Benatar M. Enteral tube feeding for amyotrophic lateral sclerosis/motor neuron disease. *Cochrane Database of Systematic Reviews* 2011(1):CD004030.
10 Stavroulakis T, Baird WO, Baxter SK, et al. The impact of gastrostomy in motor neurone disease: challenges and benefits from a patient and carer perspective. *BMJ support* 2016;6(1):52-9.
11 ProGas Study Group. Gastrostomy in patients with amyotrophic lateral sclerosis (ProGas): a prospective cohort study. *Lancet Neurol* 2015;14(7):702-9.
12 National Institute for Health and Care Excellence (NICE). Motor neurone disease: assessment and management 2016 [Available from: https://www.nice.org.uk/guidance/ng42/chapter/recommendations#nutrition-and-gastrostomy Access date: June 2019]
13 Motor Neurone Disease Association. Living with MND: Swallowing, eating and drinking. 2019 [Available from: https://www.mndassociation.org/about-mnd/living-with-mnd/eating-and-drinking/ Access date: June 2019].
14 International Patient Decision Aid Standards (IPDAS) Collaboration. What are patient decision aids 2017 [Available from: http://ipdas.ohri.ca/what.html Access date: June 2019].
15 Hogden A, Greenfield D, Caga J, et al. Development of patient decision support tools for motor neuron disease using stakeholder consultation: a study protocol. *BMJ Open* 2016;6(4).
16 Alden DL, Friend J, Schapira M, et al. Cultural targeting and tailoring of shared decision making technology: a theoretical framework for improving the effectiveness of patient decision aids in culturally diverse groups. *Soc Sci Med* 2014;105:1-8.
17 Greenaway LP, Martin NH, Lawrence V. Accepting or declining non-invasive ventilation or gastrostomy in amyotrophic lateral sclerosis: patients' perspectives. *Journal of Neurology* 2015;262:1002-13.
18 Morris MA, Dudgeon BJ, Yorkston K. A qualitative study of adult AAC users' experiences communicating with medical providers. *Disabil Rehabil Assist Technol* 2013;8:472-81.
19 NHS England. Shared Decision Making [Available from: https://www.england.nhs.uk/shared-decision-making/ Access date: June 2019]
20 NHS England. Universal personalised care [Available from: https://www.england.nhs.uk/personalisedcare/upc/comprehensive-model/ Access date: June 2019]
21 NHS. The NHS Long Term Plan. 2019 [Available from: www.longtermplan.nhs.uk Access date: June 2019]
22 NHS Confederation. Taking the value-based agenda forward: The five essential components of value-based approaches to health and care. 2018 [Available from: https://www.nhsconfed.org/resources/2018/09/taking-the-value-based-agenda-forward Access date: June 2019]
23 Patton MQ. Qualitative Research and Evaluation Methods. Thousand Oaks, CA: Sage Publications; 2002.
24 Elwyn G, Kreuwel I, Durand MA, et al. How to develop web-based decision support interventions for patients: a process map. *Patient Educ Couns* 2011;82(2):260-5.
25 Elwyn G, et al on behalf of the International Patient Decision Aids Standards (IPDAS) Collaboration. Developing a quality criteria framework for patient decision aids: online international Delphi consensus process. *BMJ* 2006;333:417.
26 Medical Research Council. Developing and evaluating complex interventions: new guidance. *BMJ* 2008;337.
27 Centre for Reviews and Dissemination. Systematic reviews: CRD's guidance for undertaking reviews in health care 2009 [Available from: https://www.york.ac.uk/media/crd/Systematic_Reviews.pdf Access date: June 2019]
28 Reyna V. A Theory of Medical Decision Making and Health: Fuzzy Trace Theory. Med Decis Making 2008;28(6):850-865.

For peer review only

29 Paynter C, Cruice M, Mathers S, et al. Communication and cognitive impairments and health care decision making in MND: A narrative review. J Eval Clin Pract 2019 Jul Epub ahead of print
30 Hobson E, Fazal S, Shaw P, et al. "Anything that makes life's journey better." Exploring use of digital technology by people living with motor neurone disease. Amyotroph Lateral Scler Frontotemporal Degener 2017;18(5-6):378-387.
31 Grimmett C, Brooks C, Recio-Sauncedo A, et al. Development of Breast Cancer Choices: a decision support tool for young women with breast cancer deciding whether to have genetic testing for BRCA1/2 mutations. Support Care Cancer. 2019;27(1):297-309.
32 Francis J, Johnston M, Robertson C, et al. "What is an adequate sample size? Operationalising data saturation for theory-based interview studies." Psychology & health 2010;25(10):1229-1245.
33 Gale N, Heath G, Cameron E, et al. Using the framework method for the analysis of qualitative data in multidisciplinary health research. *BMC Med Res Methodol* 2013;13:117.
34 DSDM Agile Project Framework Handbook. MoSCoW Prioritisation. [Available from: https://www.agilebusiness.org/content/moscow-prioritisation Access date: June 2019]
35 Yardley L, Morrison L, Andreou P, et al. Understanding reactions to an internet-delivered health-care intervention: accommodating user preferences for information provision. *BMC Med Inform Decis Mak* 2010;10(1):52.
36 NHS Health Research Authority. Consent and Participant Information Guidance: Principles of consent: General principles and role of Participant Information Sheets [Available from: http://www.hra-decisiontools.org.uk/consent/principles-general.html Access date: June 2019]

For peer review only
